# Learning by observing: a systematic exploration of modulatory factors and the impact of observationally induced placebo and nocebo effects on treatment outcomes

**DOI:** 10.3389/fpsyg.2024.1293975

**Published:** 2024-04-18

**Authors:** Helena Klauß, Angelika Kunkel, Diana Müßgens, Jan Haaker, Ulrike Bingel

**Affiliations:** ^1^Department of Neurology, Center for Translational Neuro-and Behavioral Sciences (C-TNBS), University Medicine Essen, University of Duisburg-Essen, Essen, Germany; ^2^Department of Systems Neuroscience, University Medical Center Hamburg-Eppendorf, Hamburg, Germany

**Keywords:** observational learning, social learning, placebo effect, nocebo effect, expectation, treatment outcomes

## Abstract

**Introduction:**

Observational learning (OL) refers to learning through observing other people’s behavior. OL has been suggested as an effective and simple tool to evoke treatment expectations and corresponding placebo and nocebo effects. However, the exact mechanisms by which OL shapes treatment outcomes, its moderating factors and possible areas of application remain unclear. We thus reviewed the existing literature with two different literature searches to answer the following questions: Which influencing factors contribute to OL-induced placebo and nocebo effects (in healthy volunteers and patients) and how large are these effects (search 1)? In which medical fields has OL been used so far to modulate treatment expectancy and treatment outcomes in patients, their caregivers, and at-risk groups (search 2)? We also aimed to explore whether and how the assessment of treatment expectations has been incorporated.

**Methods:**

We conducted two independent and comprehensive systematic literature searches, both carried out on September 20, 2022.

**Results:**

We identified 21 studies that investigated OL-mediated placebo and nocebo effects for pain and itch, the (placebo) efficacy of sham treatment on anxiety, and the (nocebo) induction of medication side effects (search 1). Studies showed that OL can efficiently induce placebo and nocebo effects across different presentation modes, with medium effect sizes on average: placebo effects, *d* = 0.79 (range: *d* = −0.36–1.58), nocebo effects, *d* = 0.61 (range: *d* = 0.04–1.5). Although several moderating factors have been investigated, their contribution to OL-induced effects remains unclear because of inconsistent results. Treatment expectation was assessed in only four studies. Regarding medical applications of OL (search 2), we found 12 studies. They showed that OL was effectively applied in preventive, therapeutic and rehabilitative interventions and that it was mainly used in the field of psychosomatics.

**Discussion:**

OL effects on treatment outcomes can be both positive and negative. Future research should investigate which individuals would benefit most from OL and how OL can be implemented most effectively to induce placebo and avoid nocebo effects in clinical settings.

**Systematic review registration:**

This work was preregistered at the Center for Open Science as open-ended registration (doi: 10.17605/OSF.IO/FVHKE). The protocol can be found here: https://archive.org/details/osf-registrations-fvhke-v1.

## 1 Introduction

Observational learning (OL) describes the process of acquiring knowledge and skills by observing the behavior of others. More precisely, it involves the exposure of an observer to a demonstrator model, who is presented with stimuli at a specific time point, which results in a detectable change of the model’s behavior (Heyes, 1994). OL allows individuals to learn from the consequences of the actions of others, instead of experiencing them firsthand (Fryling et al., 2011; Haaker et al., 2017). OL as a learning process was originally defined by Bandura in the context of the social learning theory (SLT) (Bandura and Walters, 1963; Bandura and Jeffrey, 1973). The initial aspects of the SLT already suggest that changes in behavior result from interactive effects of associative and cognitive influences (Bandura and Walters, 1977). The terms *observational learning*, *social learning*, *vicarious learning*, *social modeling*, and *social observation* are often used synonymously (Bajcar and Bąbel, 2018). For methodological clarity, we will use the term OL in this review. In addition to affecting an individual’s behavior (Bandura, 1986), OL can shape various cognitive processes such as the formation of beliefs, attitudes (Goubert et al., 2011), and expectations. In the medical context, patients’ expectations regarding a specific treatment are of particular interest because they can significantly impact treatment outcomes. This phenomenon is commonly referred to as the placebo or nocebo effect (Colloca and Miller, 2011a,b; Laferton et al., 2017; Bingel, 2020). Placebo and nocebo effects describe the favorable and unfavorable responses respectively, to physically and pharmacologically inert treatments. The impact of treatment expectations on health outcomes is not limited to placebo treatments such as placebo pills. Expectations can also modulate responses to active (pharmacological) treatments (Colloca et al., 2004; Bingel et al., 2011; Kirchhof et al., 2018) and even surgical interventions (Rief et al., 2017). For comprehensive reviews on treatment expectations and their impact on health and treatment outcomes, refer to Schedlowski et al. (2015) and Bingel (2020). Changing such expectations towards placebo or active treatments through classical conditioning has been shown to effectively impact treatment outcomes (Kirchhof et al., 2018; Skvortsova et al., 2020; Bajcar et al., 2021; Schwartz et al., 2022b). However, it can be challenging to implement classical conditioning in clinical care, particularly if no immediately effective treatment is at hand, as, e.g., in the case of chronic pain conditions. Therefore, it is worthwhile to identify other routes to modulate treatment expectations in clinical practice. Here, OL could serve as a potentially useful and readily accessible route in the medical context. The increasing interest in OL in the context of medical treatments, as evident from the growing number of publications on this topic in the last decade, is likely to be driven by some intriguing findings: OL- induced placebo effects have been shown to be similar in magnitude to those induced by conditioning and larger than those induced by verbal suggestions (Colloca and Benedetti, 2009), to occur with inert or placebo medications when they are labeled as a specific medication, e.g., a beta-blocker (Faasse et al., 2017), and to modulate the placebo effect and enhance the nocebo effect not only in self-reported side effects but also in physiological measures (Faasse et al., 2015). Moreover, OL interventions are relatively easy to implement (i.e., non- invasive), economical, highly standardized and, importantly, scalable (e.g., with video-based material). Furthermore, it is of significant practical importance to be aware of OL-induced nocebo effects. In situations where patients lack personal knowledge and experience with upcoming examinations and treatments, the observation of other patients can serve as an important source of information. If, e.g., a patient observes someone who claimed that a treatment was not effective or caused severe side-effects, that patient might form negative expectations and be more likely to experience nocebo effects. Hence, OL not only has the potential to positively influence patients but can also trigger negative treatment expectations and fuel fears. Thus, even brief encounters, such as in hospital waiting areas, can be crucial situations in which nocebo effects can occur through OL. Therefore, it is important to understand the conditions and factors that modulate OL in treatment contexts, so that placebo effects can be utilized systematically, and nocebo effects can be avoided (Schwartz et al., 2022b). Based on the literature, we identified the following factors that may modulate OL efficacy and are thus of particular interest for this systematic review:

### 1.1 Modulatory factors in OL efficacy

#### 1.1.1 Transmission of the OL content

##### 1.1.1.1 Observation mode/presentation mode

Observational learning is either studied in face-to-face encounters (e.g., Colloca and Benedetti, 2009; Swider and Babel, 2013; Hunter et al., 2014) [we refer to this mode of observation or presentation here as “live” or “in person” (OLp)] or by video presentation (OLv) (e.g., Hunter et al., 2014; Bieniek and Babel, 2021), in keeping with the advance of digitalization. Since video presentation is a practicable, reproducible, and therefore easily standardizable way to present learning content, as has already been shown for motor learning content (Rohbanfard and Proteau, 2013), it also appears to be suitable for observational learning in clinical use. Of particular interest in this review are, therefore, possible differences between OLp and OLv, especially regarding their effectiveness (i.e., Hunter et al., 2014).

#### 1.1.2 Observer and model characteristics

##### 1.1.2.1 Sex/gender

Recently, awareness of sex and gender differences in medicine has grown and it is also being studied in placebo and nocebo effects. For placebo analgesia, different underlying psychophysiological mechanisms in women versus men are being discussed (Dumais and Veenema, 2016; Zhang et al., 2021; Shafir et al., 2022) and symptom reports seem to vary depending on gender (Shafir et al., 2022). For example, a study investigating mass psychogenic illness revealed that women exhibit stronger tendencies to experience and articulate symptoms than men, when observing a female model (Lorber et al., 2007). Additionally, it has been suggested that sex differences may vary depending on symptoms (e.g., women may exhibit greater susceptibility to placebo effects for nausea and men for pain) and that these differences tend to be more pronounced in experimental trials than in randomized controlled trials (RCTs) (Enck and Klosterhalfen, 2019). One goal of this review is thus to summarize the findings and to complement the theories on sex differences in OL.

##### 1.1.2.2 Characteristics, traits and states of the observer

Conditioned modulation of pain, which is one of the basic mechanisms of placebo effects, has been found to be influenced by individual differences, including age, gender (see *Characteristics affecting the perception of a model*), ovulatory phase, as well as pain catastrophizing (Hermans et al., 2016), even though the evidence for the latter is mixed. Specific observer characteristics or personality traits that are thought to be relevant factors in the context of OL-induced placebo and nocebo effects are empathy (Goubert et al., 2011; Rak et al., 2013) and anxiety. Placebo effects are hypothesized to be mediated by the reduction of negative emotions, such as anxiety (Lundh, 2000), while anticipatory anxiety, manifested as autonomic arousal, has been linked to the persistence of nocebo hyperalgesia (Colagiuri and Quinn, 2018). Specifically, “fear of pain,” i.e., pain that is perceived as threatening and leads to avoidance (Vlaeyen et al., 2016), which increases stress levels and negative emotions, can reduce placebo effects (Lyby et al., 2010), and thus modulate individual susceptibility to placebo effects.

##### 1.1.2.3 Characteristics affecting the perception of a model

Beside sex and gender, model characteristics such as expertise (Lirgg and Feltz, 1991; Rakoczy et al., 2009), appearance, and status, are thought to co-determine the perception of a potential model as a reliable source of information. Consequently, these characteristics are hypothesized to mediate learning success in social learning contexts. Furthermore, given the fact that individuals can distinguish between confident and insecure role models even at a very young age (Moore et al., 1989; Rakoczy et al., 2009), the perceived self-confidence of a model may affect OL learning outcomes.

##### 1.1.2.4 Relationship between model and observer

There is evidence that the attractiveness of a model and perceived similarity between model and observer increase the effects of OL (Makuch et al., 2011). The role of perceived similarity is supported by the theoretical connotation of modeling to social comparison, a process where people observe others and use this information for self-evaluation and comparison (Wheeler and Suls, 2005). Further, the relationship and familiarity between model and observer are thought to be crucial in observational learning (Rakoczy et al., 2009) and can affect the magnitude of the neural response when observing other people’s errors (Kang et al., 2010). Previous studies have shown that perceived similarity of members within groups is higher than between groups, even if group members are assigned randomly (Simon and Pettigrew, 1990). Thus, group affiliation, perceived similarity, and the relationship between model and observer might influence OL induced effects.

#### 1.1.3 Preconditions and modifying factors

##### 1.1.3.1 Attention, memory, and awareness

Bandura and Jeffrey (1973) described attention and retention as prerequisites for OL. Here, we will present recent findings and experimental methods that explore and support this hypothesis. Regarding the role of explicit expectations, we will present results on the role of conscious versus nonconscious perception in OL and expectation formation.

##### 1.1.3.2 Expectation

Expectations are understood as a key mechanism underlying placebo and nocebo effects (Colloca and Miller, 2011a,b; Bajcar and Bąbel, 2018; Bingel, 2020). However, the most effective methods for inducing positive expectations and the extent to which stronger expectations correlate with more pronounced placebo and nocebo effects, have yet to be fully elucidated (Meeuwis et al., 2023; Rooney et al., 2023).

### 1.2 Measures of OL efficacy

#### 1.2.1 Magnitude of effects

In studies investigating a subjective perception (e.g., pain, itching or anxiety), the magnitude of an effect usually refers to the difference in ratings on a numerical rating scale (NRS) before and after an intervention. In some studies, also objective measures such as blood pressure (BP), heart rate (HR) and skin conductance are assessed. Previous research has focused on assessing the magnitude of different acquisition ways that can generate placebo and nocebo effects, indicating that conditioning can produce larger effects than verbal instructions, and that the combination of both mechanisms can yield stronger effects than either mechanism alone (Vase et al., 2002; Petersen et al., 2014; Wolters et al., 2019). Here, we will give an overview of the range of effect sizes that can be achieved with different types of OL.

#### 1.2.2 Temporal dynamics of effects

The temporal dynamics of placebo and nocebo effects are usually tested by observing changes in subjects’ ratings over the course of an experiment. Some studies also include follow-up observations, in which the primary outcome is not only tested directly after an intervention but at a later time point. Previous research has demonstrated that placebo analgesia, whether induced through conditioning or OL, can persist (does not extinguish) over time (Colloca and Benedetti, 2009; Egorova et al., 2015). However, the stability of effects is influenced by the type of conditioning, with partial reinforcement yielding more favorable effects than continuous reinforcement (Au Yeung et al., 2014), and by the total number of conditioning trials (Colloca et al., 2010). If OL could produce durable and lasting treatment benefits, it would be an ideal candidate for clinical applications.

### 1.3 Objectives

Despite numerous studies demonstrating the effectiveness of OL in inducing placebo and nocebo effects (Colloca and Benedetti, 2009; Faasse and Petrie, 2016; Bajcar and Bąbel, 2018), the exact mechanisms through which these effects can be targeted and maximized are not yet fully understood.

We will build upon the findings of two recent systematic reviews and a meta-analysis (Meeuwis et al., 2023) on observationally induced placebo hypoalgesia (Schwartz et al., 2022b) and nocebo hyperalgesia (Meeuwis et al., 2023), and extend the scope to include symptoms other than pain to understand how OL can be transferred to a variety of clinical applications. Furthermore, we extent a systematic review investigating placebo effects on cutaneous pain and itch (Blythe et al., 2023) by including nocebo studies and restricting the results to OL.

In order to further characterize the transfer of OL to clinical application, the second purpose of this review is to provide an overview of the effect of OL in health-related outcomes in RCTs. Whereas search 1 was focusing on experimental effects of OL in studies that were often designed to examine or manipulate the factors contributing to OL, the intention of this second search was to characterize the clinically relevant fields that could successfully apply OL within the medical regimes in a well-controlled RCTs. Search 2 thereby examines applicability of OL in the medical context and provides information on how OL can be translated from experimental work to clinically established treatments.

The objective of this systematic review is thus threefold: (I) to provide an overview of studies that have used OL to induce placebo and nocebo effects, (II) to identify factors, including experimental factors (e.g., presentation mode) that may modulate whether and how the observation of treatment effects in others impacts placebo and nocebo effects, and (III) to draw conclusions on how OL-based interventions can be applied to enhance health-related clinical outcomes. To address these research questions, we conducted two independent systematic literature searches:

Search 1 was conducted to identify studies that examined if OL can induce placebo and nocebo effects in experimental conditions as well as in clinical situations, how large these effects are and how long they persist. We searched for studies that provided information on the following questions: Do OL-induced placebo and nocebo effects correlate with expectations? Which observer and model characteristics are relevant to or even prognostic for these effects and does the relationship between model and observer affect the outcome? Further, we are interested if the presentation mode of an observation (live vs. videotaped) influences the magnitude of OL-induced effects.

Search 2 was conducted to identify and review clinical trials of patients and their caregivers to determine the medical areas in which OL has been studied and applied to date and to capture its impact on treatment expectancy and outcome.

## 2 Methods

This systematic review followed the Preferred Reporting Items for Systematic Reviews and Meta- Analysis (PRISMA; Page et al., 2021) guidelines and consists of two independent systematic literature searches, both carried out on September 20, 2022. It was preregistered at the Open Science Framework (doi: 10.17605/OSF.IO/FVHKE). We queried the databases PubMed, Web of Science, Scopus, and PsycINFO. Full search terms can be found in the supplements (Supplementary Table S1).

### 2.1 Article selection and data collection process

#### 2.1.1 Search 1

**Figure 1:**
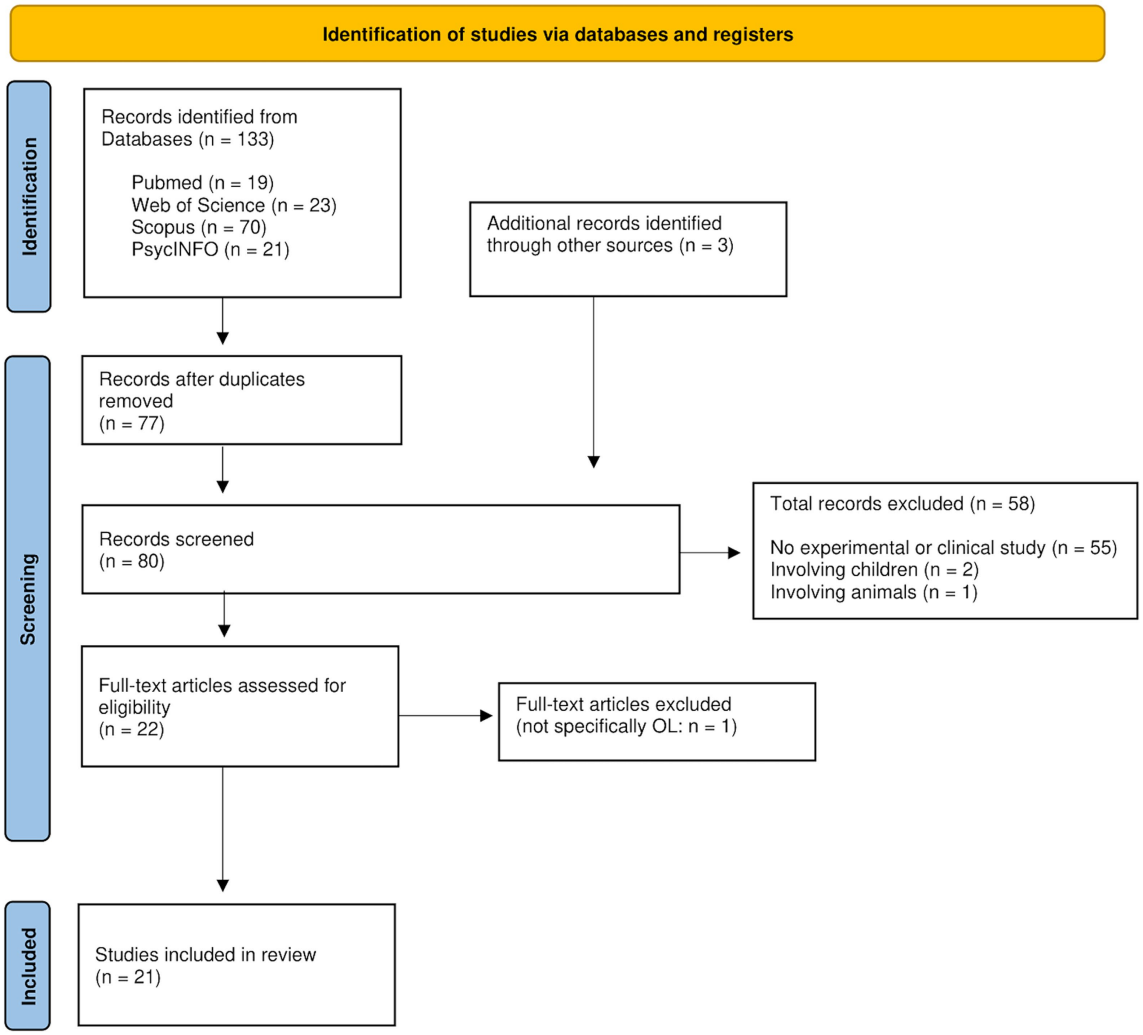
PRISMA flow diagram for data selection process modified from Page et al. (2021) for the first search. Records were identified from databases by the application of search queries (see Supplementary materials). After screening and evaluating, 21 eligible studies were included in this systematic review.

We included experimental and clinical studies about placebo and/or nocebo effects, and/or (treatment) expectation. As search terms, we used fixed terms such as “placebo response” and “placebo effect” to avoid confusion with other meanings of the term “placebo,” such as pharmacologically inactive drugs or placebo control groups in drug trials. The term “model” refers to the person whose behavior is being observed, while “participant” or “observer” refers to the subject participating in the study who is observing the model. We excluded studies on animals to better compare our results with human therapeutical settings. We also excluded studies with children because they differ from adults in brain structure, neural plasticity, and (visual perceptual) learning processes (Frank et al., 2021). Following the PRISMA guidelines, we evaluated the search results in a stepwise manner (Figure 1). First, we scanned all obtained hits to gain a full comprehensive picture of the scope of studies investigating OL in medicine to date. Moreover, considering all hits, including reviews, helped us to identify important primary literature. Second, we excluded reviews and selected only suitable experimental and clinical studies. Since eligibility of all results was evaluated independently by two authors (HK, AK), we were able to achieve interrater reliability. Only studies deemed eligible by both authors were included (Table 1).

**Table 1:** Results of the first search.

Authors and year of publication	Topic	Aim of the study	Primary target outcome	Design	Participating observers (*N*, sample, mean age, sex)	Observed models (sex)	Type of placebo agent/Intervention	Presentation mode (OLp/OLv)	OL effect size and interpretation of effect size (Cohens *d*)
Faasse et al. (2018)^1^	Treatment side effects	To investigate the impact of social modeling of side effects on symptoms following placebo treatment, the role of participant and model gender, and trait viewer empathy.	Symptoms and intensity	PG	96^HP^ (21 y/o) 48 ♂ 48 ♀	♀ and ♂	Sham Modafinil nasal spray (isotonic saline solution)	OLp	*d* = 0.56^m^
Swider and Babel (2013)^2^	Nocebo hyperalgesia	To study the effect of sex of both the model and the subject on placebo analgesia induced by observational learning, to replicate the analgesic placebo effect by observational learning discovered by Colloca and Benedetti (2009) and to study the effect of the color of light stimuli on pain ratings.	Pain intensity (electric pain), empathy	PG	84^HP^ (23 y/o) 42 ♂ 42 ♀	♀ and ♂	Light stimuli	OLp	η^2^ = 0.36 (*d* = 1.50)^l^
Faasse et al. (2015)^3^	Treatment side effects	To investigate the impact of the social modeling of side effects on both the nocebo effect and the placebo effect following the administration of a placebo tablet described as a beta-blocker medication. To investigate the role of medication branding in moderating social modeling effects.	Differences in symptom reporting, blood pressure, heart rate, and anxiety	PG	82^HP^ (21 y/o) 42 ♀ 41 ♂	♀	Sham Betablocker pill (placebo tablet)	OLp	Number of total symptoms: η_p_^2^ = 0.10 (*d* = 0.67)^m^ Number of symptoms attributed to side effects: *η_p_^2^* = 0.09 (*d* = 0.63)^m^
Faasse et al. (2017)^4 P^	Treatment effectiveness	To investigate the impact of the social modeling of medication benefits on placebo treatment effectiveness following the administration of an inert tablet described as a beta-blocker.	Heart rate, anxiety	PG	59^HP^ (28 y/o) 35♀ 24♂	♀	Sham Betablocker pill (placebo tablet)	OLp	Heart rate: *d* = 0.63^m^ Anxiety: *d* = 0.46^m^ (trend) Systolic blood pressure: *d* = 0.51^m^ (trend)
Hunter et al. (2014)^5^	Placebo analgesia	To compare the effect of social learning through video recording versus live observation of a demonstrator. To investigate whether the relationship between analgesic response and empathy traits is similar in both conditions.	Pain intensity (electric pain), empathy trait	PG	60^HP^ (27 y/o) 60♀	♀	Light stimuli	OLp and OLv	OLv: *r* = 0.59 (*d* = 1.46)^l^ OLp: *r* = 0.62 (*d* = 1.58)^l^
Brączyk and Bąbel (2021)^6^	Placebo analgesia	To investigate the effect of a model’s self-confidence and the observer’s self-esteem and self-efficacy on observationally acquired placebo analgesia.	Pain intensity (electric pain), psychological traits	PG	60^HP^ (24 y/o) 36♀ 24♂	♂	Colour stimuli	OLv (Duration of the video: 4:00 min and 2:58 min)	Self-confident model (SCM): *η^2^* = 0.10 (*d* = 0.67)^m^ Un-self-confident model (UCM): *η^2^* = 0.12 (*d* = 0.74)^m^
Egorova et al. (2015)^7*^	Pain perception and placebo/nocebo effects	To examine the effect of subliminally and supraliminally presented conditioned pain cues, established using both, conditioning and observational learning procedures, on pain perception and placebo/nocebo effects.	Pain intensity (heat pain), skin conductance	WS	20^HP^ (23 y/o) 12♀ 8♂	♀ and ♂	Visual stimuli	OLv	*n.r.*
Vögtle et al. (2016)^8^	Nocebo hyperalgesia	To investigate socially induced nocebo effects and possible mediators of socially induced nocebo hyperalgesia, such as pain catastrophizing, somatic complaints, hypochondriacal concerns, and empathy, in a sample from the general population.	Pain intensity (pressure pain)	PG	97^HP^ (43 y/o) 97♀	♀	Hypoallergenic ointment	OLv (Duration of the video: 10 min. 3 s.)	*d* = 0.44^m^
Vögtle et al. (2013)^9^	Nocebo hyperalgesia	To investigate whether a nocebo response can be induced by verbal suggestion as well as by social observational learning.	Pain intensity (pressure pain)	PG	80^HP^ (23 y/o) 80♀	♀	Hypoallergenic ointment	OLv (Duration of the video: 10:22 min)	*d* = 0.52^m^
Zhang et al. (2017)^10*^	Placebo analgesia and nocebo hyperalgesia	To examine the sustained effect of prior experience induced by social observation on placebo/nocebo responses to subsequent treatment.	Pain intensity (electric pain)	PG	82^HP^ (21 y/o) 82♀	♂	Metal ring (sham device)	OLv	*n.r.*
Bajcar et al. (2020)^11^	Placebo analgesia	To investigate whether introducing the model as either another participant taking part in the study, or a co-worker of the experimenter would affect the magnitude of observationally induced placebo analgesia. To clarify previous results by investigating the effects of empathy, conformity, and fear of pain on the magnitude of the placebo effect induced by OL.	Pain intensity and pain expectancy (electric pain)	PG	96^HP^ (22 y/o) 60♀ 36♂	♀	Light stimuli	OLp	Demon-strator group: *η_p_^2^* = 0.12 (*d* = 0.74)^m^ Co-participant group: *η_p_^2^* = 0.19 (*d* = 0.97)^l^
Schenk and Colloca (2020)^12^	Placebo hypoalgesia	To investigate the neural processes of observational learning, as well as OL-induced placebo hypoalgesia.	pain intensity, pain unpleasant-ness, pain expectation (heat pain)	WS	38^HP^ (28y/o) 23♀ 15♂	♂	Visual cues, cream (colored skin lotion)	OLv (Duration of the video: 11–13 s)	*n.r.*
Blythe et al. (2021)^13(*)^	Nocebo effect on itch	To investigate the efficacy of classical conditioning and OL for inducing nocebo effects on cowhage-evoked itch and scratching behavior.	Itch intensity	PG	58^HP^ (22 y/o) 58♀	♀	Inert gel	OLv (Duration of the video: 38:03 min)	*n.s.*
Świder and Bąbel (2016)^14^	Placebo analgesia	To investigate the effect of the type and color of placebo stimuli on the placebo effects induced by OL. To investigate the effects of empathy and both pain anxiety and fear of pain on the magnitude of the placebo effects induced by OL.	Pain intensity (electric pain)	PG	65^HP^ (22 y/o) 65♀	♀	Light stimuli, visual stimuli	OLp	*η^2^* = 0.24 (*d* = 1.12)^l^
Colloca and Benedetti (2009)^15(*)^	Placebo analgesia	To investigate the role of OL in placebo analgesia in a human experimental setting, whereby subjects learn by observing the analgesic experience of others.	Pain intensity (electric pain), heart rate	PG	48^HP^ (23 y/o) 48♀	♂	Sham electrode, light stimuli	OLp	*n.r.*
Vögtle et al. (2019)^16^	Nocebo hyperalgesia	To investigate whether observing natural pain behavior, such as facial pain expressions, can also induce nocebo responses.	Pain intensity (pressure pain)	PG	80^HP^ (22 y/o) 80♀	♀	Inert ointment	OLv (Duration of the video: 9 min 19 s and 9 min 33 s)	*n.s.*
Bieniek and Bąbel (2021)^17^	Placebo analgesia	To determine whether OL-induced placebo analgesia can be influenced by the social status of a model.	Pain intensity (electric pain)	PG	60^HP^ (23 y/o) 32♀ 28♂	♂	Light stimuli	OLv	High status model group: η^2^ = 0.12 (*d* = 0.74)^m^ Low status model group: *η^2^* = 0.07 (*d* = 0.55)^m^ Difference between groups: *n.s.*
Schwartz et al. (2022)^18+^	Augmented therapeutic placebo effect	To test if observing positive drug effects on pain and mobility in another patient could increase pain reduction and functional capacity in chronic low back pain patients.	Pain intensity	PG	44^CBP^ (63 y/o) 27♀ 17♂	♂	Model observation, analgetic dosage of Amitriptyline (medication intake started before study)	OLp	Pain reduction: *n.s.* Functional capacity: *d* = 0.63^m^
Raghuraman et al. (2019)^19^	Placebo hypoalgesia	To determine the neurophysiological changes associated with pain relief acquired through observation by using EEG and to understand how and when the brain responds to observationally induced placebo hypoalgesia.	Pain intensity (heat pain)	WS	31^HP^ (23 y/o) 19♀ 12♂	♂	Hypoallergenic cream (coloured)	Pictures	*d* = 0.36^s^
Buglewicz-Przewoźnik et al. (2022)^20^	Allodynia	To test whether OL alone can elicit allodynia, if allodynia induced by OL is stronger when the time between observation and stimulation is shorter, and if allodynia is stronger when a pain-maximizing model is present (*post hoc* +) during stimulation of the observer.	Pain intensity (electric pain)	PG	88^HP^ (24 y/o) 44♀ 44♂	♂	Model observation	OLp	Significant difference in risk of experiencing pain (RR): Real time vs. control group: RR: 3.83 (95% CI: from 3.01 to 4.91, *p* < 0.001) *post hoc+* group vs. control group: RR: 3.28 (95% CI: from 2.56 to 4.23, *p* < 0.001) *post hoc*- group vs. control group: RR: 2.93 (95% CI: from 2.28 to 3.80, *p* < 0.001)
Tu et al. (2019)^21*^	consciously and non- consciously conditioned placebo and nocebo effects	To investigate the neural pathways of conditioning and observational learning for conscious and nonconscious conditioned placebo/nocebo effects using MEG.	Pain intensity (heat pain)	WS	21^HP^ (25 y/o) 12♀ 9♂	♀ and ♂	Visual cues	OLv	*n.r.*

**Figure 2:**
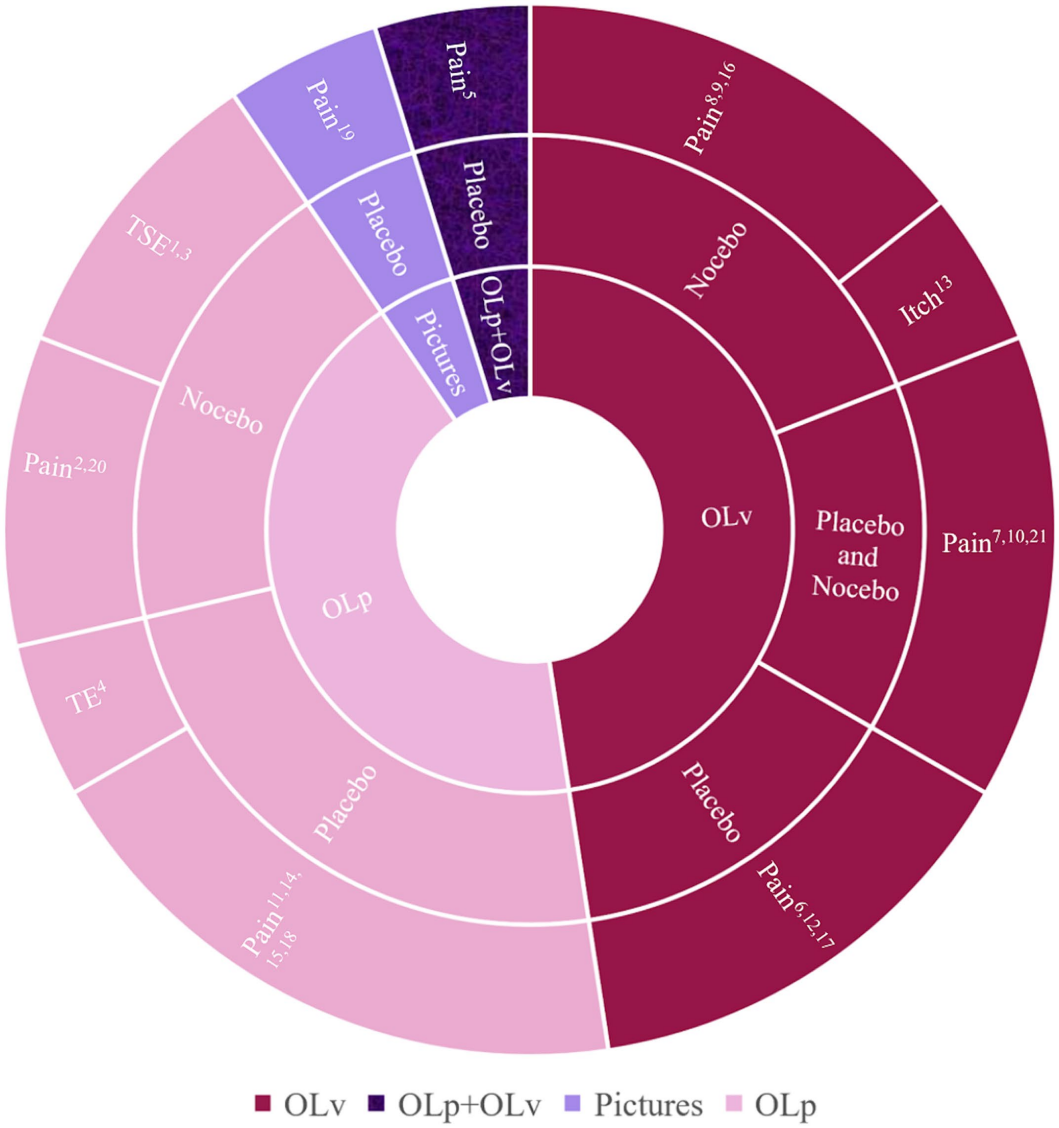
Results from search 1 (*n* = 21) categorized by observation mode tested with OLv (*n* = 10) and OLp (*n* = 9) (inner circle), placebo/nocebo condition (middle circle), and medical condition (outer circle). Please refer to Table 1 containing all the references. OL, observational learning; OLp, OL in person; OLv, OL via video; TE, treatment effectiveness; TSE, treatment side effect.

Studies included in the first search. Author names, year of publication, study aims, and the characteristics of the participating observers and the observed models are presented. Stimuli and Intervention (how placebo effects were elicited in each study), presentation mode (live models or video recordings), and effect sizes of each study are listed. If not indicated otherwise, all studies involved healthy volunteers. For details on the interpretation of effect sizes please see Cohen (1988) and Lakens (2013). Superscript numbers indicate references for Figures 2 and 5. ^+^Conducted on patients. ^*^Observational learning with repeated stimulus presentation. ^(*)^Studies comparing conditioning to OL; ^P^pilot study; observing participants: HP, healthy patients; CBP, chronic back pain patients; effect sizes: s, small; m, medium; l, large. OL, observational learning; OLp, observational learning in person (live, face-to-face); OLv, observational learning by video; PG, parallel group design; WS, within subject design; d, Cohens *d*; r, Pearson’s *r*; η^2^ = eta square; ηp^2^, partial eta square; RR, relative risk; n.r., not reported; n.s., not significant.

#### 2.1.2 Search 2

We included RCTs conducted with patients, people at health risk, relatives of patients, and caregivers. Analogously to the first search, we excluded studies on animals. However, we did not exclude studies with children, as our second search covered a broader research question and was intended to reflect the actual areas of application.

We preselected to include (pilot) RCTs in the search mask and excluded duplicates. Since OL was often used as part of a multimodal therapeutic approach, without specific description of the OL intervention or reports of independent OL effects, we only included studies that gave more detailed descriptions of the OL procedures. Again, all hits were evaluated independently by two authors and only studies on which both authors agreed were included (Supplementary Table S2).

### 2.2 Data items and synthesis method

#### 2.2.1 Search 1

We scanned the eligible articles for the following information: medical field, details about study participants (age, gender), type of trial, details about the observed model (age, gender), experimental design, cues, experimental stimuli, treatment, intervention, measurements, attention control, observation mode, and effect sizes. For the effect sizes, we extracted all reported effect sizes from the included articles. We specifically focused on effect sizes that described the observational learning effect on the placebo or nocebo effect. If other effect sizes were reported (η^2^, η_p_^2^, relative risk (RR)) we converted them into Cohen’s d to facilitate comparability. Comprehensive details regarding the reported effect sizes can be found in Table 1. If video material was used, we requested video material from the research groups and analyzed the additional variables like setting and instructions to the participants, duration of the video, and image section.

#### 2.2.2 Search 2

RCT articles were sought for the following details: application field (prevention, therapeutical intervention, rehabilitation), addressed medical condition, intervention, observation mode, measurements, participants, duration of intervention, and positive or negative effects, including effect size. Since one article was not accessible in full length, we were only able to collect partial data from the abstract. All hits were categorized into three groups according to their medical setting: prevention, therapeutical interventions (and therapeutical targeted disease management), and rehabilitation.

### 2.3 Risk of bias assessment

Risk of bias assessment was based on the assessment of methodological quality by Downs and Black (1998) but modified with respect to suggestions by Meeuwis et al. (2023) and additional changes by the authors. Adjustments as well as results are displayed in the Supplementary Table S3 and Supplementary Figures S1–S4.

## 3 Results

### 3.1 Search 1

For the first search, a total of 21 studies were included, consisting of 20 experimental studies conducted with healthy volunteers primarily recruited from university communities, and one RCT involving chronic back pain patients. Among these studies, 17 focused on pain. Sixteen of the pain studies were experimental. In these experiments, the observation of a model experiencing either pain or pain relief, which was predicted by a cue (e.g., light), resulted in hyper- or hypoalgesia in the observer, when confronted with the same (conditioned) cue. The placebo and nocebo treatments applied, respectively, can be found in Table 1. Please note that Buglewicz-Przewoźnik et al. (2022) did not include a treatment but that the observation itself induced an allodynic effect.

The PRISMA flow diagram illustrating the study selection process is presented in Figure 1. Refer to Table 1 for a comprehensive overview of all included studies, and to Table 2 for a brief summary of the medical conditions and effects tested within these studies.

**Table 2:** Overview of search 1 results.

Effect	Medical Condition		Sum
	Pain	Other	
Placebo effect	Analgesia/Hypoalgesia (*n* = 9)	Treatment effectiveness (*n* = 1)	*n* = 10
Nocebo effect	Hyperalgesia (*n* = 4) Allodynia (*n* = 1)	Treatment side effects (*n* = 2) Itch (*n* = 1)	*n* = 8
Both	Hyperalgesia (*n* = 4) Allodynia (*n* = 1)	–	*n* = 3
Sum	*n* = 17	*n* = 4	*n* = 21

Overview of the sample of studies included in this review (*n* = 21), grouped by the effects measured (placebo effect, nocebo effect, or both) and their content (pain or other), with the respective number of included studies in parentheses.

#### 3.1.1 Magnitude of placebo and nocebo effects with OL

Ten studies investigated OL in relation to placebo effects, eight studies focused on nocebo effects, and three studies examined both placebo and nocebo effects. To measure the effect size, subjective ratings on a NRS were compared before and after the respective intervention. Additionally, some studies included objective outcome measured like BP and HR. In Table 1, respective primary target outcomes and effect sizes are summarized. Remarkably, 20 of 21 studies reported significant OL-induced effects with medium to large effect sizes on average (average effect size for placebo effects: *d* = 0.79, range: *d* = −0.36 – 1.58; average effect size for nocebo effects: *d* = 0.61, range: *d* = 0.04 – 1.5). While these averages are nominally larger for placebo effects, studies that directly compared OL-induced placebo and nocebo effects showed significantly larger nocebo than placebo effect sizes (Zhang et al., 2017; Tu et al., 2019).

##### 3.1.1.1 OL compared to conditioning and verbal instructions

Three studies directly compared the effectiveness of OL to other ways of inducing placebo and nocebo effects, i.e., verbal instructions and classical (also called “firsthand”) conditioning. Colloca and Benedetti (2009) compared all three mechanisms and found the strongest pain reduction (43.4%) for classical conditioning, 39.2% for OL, and 8.4% for verbal instructions. OL thus led to placebo effects of similar magnitude to classical conditioning. Blythe et al. (2021) found that conditioning induced nocebo effects of itch, whereas OL with verbal suggestion did not. However, the authors concluded that this lack of significant effect may have been partly influenced by the long duration of the video. Vögtle et al. (2013) reported a successfully induced nocebo response via OL but not via verbal suggestion. Additionally, Schwartz et al. (2022a) suggested that visually observing a sham patient may result in a stronger placebo effect than merely hearing a verbal report about reduced pain. Three studies examined observational learning in conditioning paradigms. While in classical conditioning, an association between a cue (e.g., a color cue) and a stimulus (e.g., a firsthand pain stimulus) is established through repeated paired presentations, here, the association is acquired by observing another person undergoing this process. The experimental designs varied among studies. In one study, participants were assigned to either the observational learning or classical conditioning group (Zhang et al., 2017). In two studies, each participant underwent both interventions sequentially (Egorova et al., 2015; Tu et al., 2019). Interestingly, classical conditioning and observational learning were found to be equally effective (Egorova et al., 2015) and to have similar influences on placebo and nocebo effects in subsequent treatments (Zhang et al., 2017). Additionally, one study reported a positive correlation between the two, indicating that direct experience and learning from others might could have a similar effect on an individual (Tu et al., 2019).

##### 3.1.1.2 Observation and presentation mode

The studies used three different presentation modes of the model: a real person (*n* = 9), videotapes (*n* = 10), or pictures (*n* = 1). See Figure 2 for an overview.

###### 3.1.1.2.1 Observational learning in person

The nine studies investigating observational learning in person (OLp) were conducted either in experimental settings (Colloca and Benedetti, 2009; Swider and Babel, 2013; Świder and Bąbel, 2016; Bajcar et al., 2020; Buglewicz-Przewoźnik et al., 2022) or in clinical settings like waiting areas (Faasse et al., 2015, 2017, 2018) and doctors’ rooms (Schwartz et al., 2022a). Participants observed a sham participant undergoing an experiment in which they later participated themselves or they met a sham patient who reported on his or her symptoms or well-being. OLp induced significant placebo/nocebo effects in all studies (see Table 1 for effect sizes).

###### 3.1.1.2.2 Video-based observational learning

Video-based observational learning (OLv) was used in 10 studies. Here, participants were asked to watch a video of a model taking part in the experiment before undergoing it themselves. The videos showed the identical experimental set-up. The models’ faces and their expressions were either fully presented (Egorova et al., 2015; Schenk and Colloca, 2020), or were only partially visible depending on the viewing angle (Vögtle et al., 2013, 2016). The duration of the videos ranged from a few seconds to 10 min. Only one study showed the whole experimental procedure, and this video lasted more than 30 min. In this experiment, observation of the conditioning procedure via video (OLv group) did not evoke significant effects while direct experience (conditioning group) led to nocebo effects. The authors concluded that the long video may have caused participants to lose interest and therefore did not evoke a significant effect (Blythe et al., 2021). The nine other studies investigating observational learning with videos found significant placebo/nocebo effects.

###### 3.1.1.2.3 Comparison of OLv and OLp

Hunter et al. (2014) compared placebo analgesia induced by OLv versus OLp and found significant effects of similar magnitude in both groups (OLp: *r* = 0.62 vs. OLv: *r* = 0.59). Furthermore, their results suggested a greater involvement of empathy for OLp than for OLv (Hunter et al., 2014), a hypothesis that is discussed in another study as well (Raghuraman et al., 2019).

###### 3.1.1.2.4 Static pictures

Only one study used static pictures and reported a successful induction of placebo effects (*d* = −0.361) (Raghuraman et al., 2019).

##### 3.1.1.3 Model and observer characteristics and traits

###### 3.1.1.3.1 Characteristics, traits and states of the observer

####### 3.1.1.3.1.1 Sex and gender

In the literature reviewed, the terms “gender” and “sex” were not clearly differentiated. Therefore, we will treat them together in the following section and refer to the terms as used in the respective study. Thirteen studies included both female and male participants who were counterbalanced across the experimental conditions. Eight studies were conducted exclusively with female participants, while no study was conducted with only male participants. Among these, one study demonstrated that female participants exhibited greater placebo effects after observing a female model (Faasse et al., 2017), while no gender differences were observed after observing a male model (Raghuraman et al., 2019). However, another study showed that greater nocebo effects were found in females compared to males, irrespective of the model’s sex (Swider and Babel, 2013). Likewise, Faasse et al. (2015) found a significant increase in side effect reports in female, but not in male participants, after observing a female model. Moreover, in one study, female participants experienced significantly more *general, non-modeled symptoms* than male participants, especially after observing a female model. However, because this result did not apply to specifically *modeled symptoms*, it cannot be concluded that women are more receptive to social modeling (Faasse et al., 2018).

####### 3.1.1.3.1.2 Empathy

Five out of 15 studies included empathy as covariate for the magnitudes of placebo and nocebo effects. Empathy was assessed with the Interpersonal Reactivity Index (IRI), which measures empathy on four subscales: perspective taking, fantasy, empathic concern, and personal distress. Empathic concern was positively correlated with placebo analgesia (Colloca and Benedetti, 2009; Bieniek and Bąbel, 2021) and nocebo hyperalgesia (Swider and Babel, 2013) and also depended on the observation mode (OLp vs. OLv) (Hunter et al., 2014). Perspective taking and personal distress were significantly predictive for side effects (Faasse et al., 2018). Nine studies however, did not find empathy to be a predictive parameter for the magnitude of observationally induced placebo or nocebo responses and found no significant correlation between stimulus ratings and empathy scores (Vögtle et al., 2013; Świder and Bąbel, 2016; Vögtle et al., 2016; Raghuraman et al., 2019; Vögtle et al., 2019; Bajcar et al., 2020; Schenk and Colloca, 2020; Blythe et al., 2021; Schwartz et al., 2022a).

####### 3.1.1.3.1.3 Covariates of pain perception: fear of pain, pain catastrophizing, individual pain thresholds/pain sensitivity

Three out of four studies that assessed fear of pain found no correlation with the magnitude of the placebo and nocebo effect (Vögtle et al., 2013; Świder and Bąbel, 2016; Bajcar et al., 2020). Only Schenk and Colloca (2020) found that participants in the placebo condition felt less anxious about the upcoming pain and showed reduced BOLD responses in the amygdala and periaqueductal grey, i.e., in brain areas associated with responses to threats and the observational acquisition of fear (Haaker et al., 2017). Vögtle et al. (2013) found a positive correlation between the magnitude of the evoked nocebo response and participants’ tendency for pain catastrophizing, particularly for the subscale helplessness. However, a significant influence of pain catastrophizing was not replicated in later studies (Vögtle et al., 2016, 2019). Raghuraman et al. (2019) found that the magnitude of observationally induced hypoalgesia neither correlated with participants’ pain tolerance nor with their individual pain thresholds. The authors concluded that placebo hypoalgesia could be induced regardless of participants’ individual pain sensitivity.

####### 3.1.1.3.1.4 Conformity and susceptibility to social influence

Results of two studies suggest that neither participants’ social conformity (Bajcar et al., 2020; Buglewicz-Przewoźnik et al., 2022), nor their tendency to yield social influence (Bajcar et al., 2020) seemed to be associated to the magnitude of OL-induced analgesia and allodynia.

####### 3.1.1.3.1.5 Self-esteem and self-efficacy

One study hypothesized that an observer’s self-esteem might influence the magnitude of an evoked effect (Brączyk and Bąbel, 2021). However, the authors found no predictive correlation between an observer’s self-esteem or self-efficacy and the evoked placebo analgetic response.

###### 3.1.1.3.2 Model characteristics

####### 3.1.1.3.2.1 Gender

The influence of a model’s gender on OL-induced effects was only investigated in two studies. Swider and Babel (2013) investigated its role in nocebo hyperalgesia. They found that for both female and male observers, the nocebo effect was more pronounced when a male model was observed compared to a female model. Based on this finding, several subsequent OL studies chose male instead of female models (Buglewicz-Przewoźnik et al., 2022). On the other hand, Faasse et al. (2018) showed that participants (regardless of gender) were significantly more likely to show objectively measurable side effects (heart rate, blood pressure) after observing a female rather than a male model, even if the overall response to social modelling did not differ significantly.

####### 3.1.1.3.2.2 Social status

One study investigated the role of social status and found the perceived social status of a model to be a significant predictor for the magnitude of placebo analgesia (Bieniek and Bąbel, 2021). However, the effect was only significant for an implicit assessment (rating) but not for the explicit questions about the model’s social status, possibly due to social unacceptability.

####### 3.1.1.3.2.3 Model’s self-confidence

Brączyk and Bąbel (2021) found a significant positive relationship between the perceived self-confidence of a model and the magnitude of the measured placebo analgesic response.

###### 3.1.1.3.3 Similarities, concordance and relationship between model and observer

####### 3.1.1.3.3.1 In-group versus out-group membership

Bajcar et al. (2020) tested the influence of the role played by a model (co-participant versus coworker of the experimenter) and the associated group affiliation but did not find this to be a significant predictor for the placebo effect.

####### 3.1.1.3.3.2 Sex/gender concordance/congruency

Two studies in which the sex of the participants and the model were matched in the experimental groups found no significant effect on the magnitude of evoked effects (Swider and Babel, 2013; Faasse et al., 2018). However, Faasse et al. (2018) reported that matched female observer and female model gender led to significantly higher rates of general symptom reports than any other combination of model and observer gender. This misattribution of symptoms as treatment side effects was more pronounced when participants sat with a model of the same gender than when they sat with a model of the opposite gender.

####### 3.1.1.3.3.3 Age congruence

Although not all studies provided detailed information about the model’s and observer’s demographics, it appears that study participants were, on average, similar in age to the respective model in that study. Eighteen studies were conducted with participants (mostly students) in their twenties (age range 20–28). Likewise, models of the respective studies were described as in their twenties or as being students themselves. In some cases, no explicit information was given but the images provided allowed for an estimate of the age of the models. Two studies included older participants, i.e., mean age = 43 years (Vögtle et al., 2016) and mean age = 63 years (Schwartz et al., 2022a), and models were described as “mid-forties” and “pensioner.” Overall, while not explicitly stated, it seems that the authors have considered the congruence in age groups between the model and the observer across all the studies.

##### 3.1.1.4 Attention, memory, and awareness

###### 3.1.1.4.1 Attention and memory

Ten studies controlled participants’ attention during the observation phase of the respective experiment by asking them to take notes (Colloca and Benedetti, 2009; Swider and Babel, 2013; Świder and Bąbel, 2016; Bajcar et al., 2020; Brączyk and Bąbel, 2021), by informing them that they would need to answer questions afterwards (Vögtle et al., 2013, 2016; Bieniek and Bąbel, 2021), or by directly asking them to memorize details (Zhang et al., 2017; Vögtle et al., 2019). Schwartz et al. (2022a) used a post-hoc assessment of participants’ explicit memory about a sham patient who entered the room during the trial. Only a few participants could recall the attendance of the sham patient after the trial, however there was no significant difference between the OL group and the control group. Raghuraman et al. (2019) used electroencephalogram (EEG) to measure event-related potentials (ERPs) during the anticipatory phase of OL placebo hypoalgesia. They analyzed the (visually provoked) P2 component, which is involved in attention processes (Lijffijt et al., 2009; Eldar and Bar-Haim, 2010) and is larger in response to threatening images compared to neutral ones (Carretié et al., 2004) and found smaller P2 amplitudes for treatment cues that were associated with larger OL-induced placebo hypoalgesia. The authors concluded that this indicates a reduced attentional engagement for treatment cues that are accompanied by greater OL placebo hypoalgesia. These results further suggest that placebo-non-responders have a higher information processing rate and more attentional engagement in response to anticipatory cues, than placebo responders.

###### 3.1.1.4.2 Awareness

Awareness here refers to the conscious versus unconscious perception of a stimulus. Two studies tested the role of awareness in OL-induced placebo and nocebo effects by modifying the presentation duration of different visual cues. Fractal images (Egorova et al., 2015), or neutral male face images (Tu et al., 2019) were presented either supraliminally, i.e., consciously perceivable, or subliminally, i.e., below the threshold for conscious perception. In both studies, supraliminally presented cues evoked significant placebo and nocebo effects in the observational learning condition. In contrast, for subliminally presented cues, either only placebo but not nocebo effects were significant (Egorova et al., 2015) or no significant effects were observed at all (Tu et al., 2019). Overall, both studies found a significant effect of awareness: conditioned placebo and nocebo effects were stronger for conscious than for non-conscious cue presentations.

**Figure 3:**
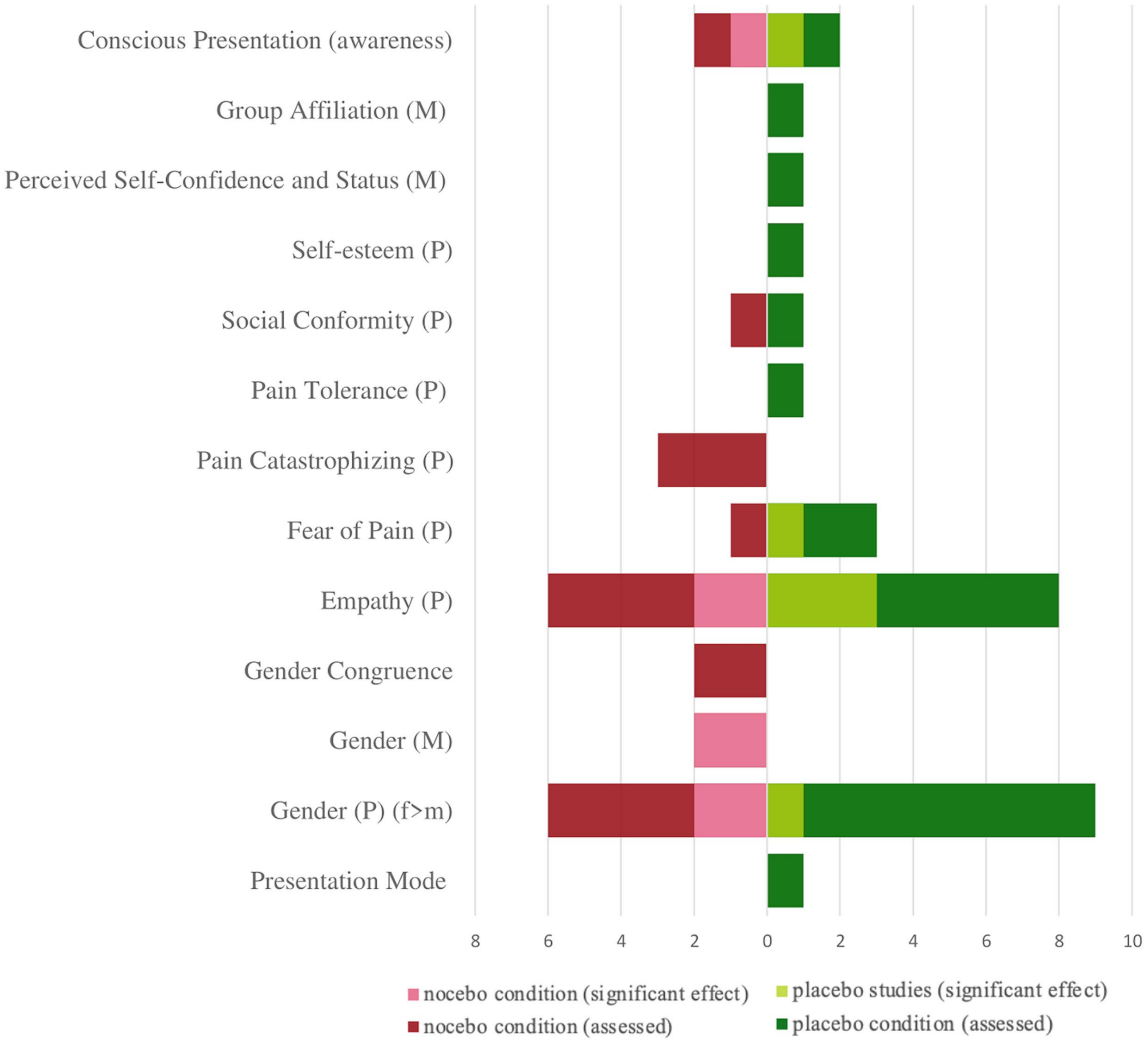
Representation of the investigated OL influencing factors (search 1) according to conditions (placebo/nocebo). Number of study conditions in which these factors were investigated and corresponding number of significant results. P, participant/observer; M, model; f, female; m, male.

Figure 3 provides an overview of the number of study-conditions that investigated the aforementioned influencing factors. Instead of listing only the individual studies, all individual study conditions were also considered here, i.e., a single study testing both placebo and nocebo conditions is listed here as two study conditions. The lighter bars indicate how many study arms investigated a given factor of placebo (right) or nocebo (left) effects, and the darker bars indicate how many of them found significant results.

##### 3.1.1.5 Expectations

Only four out of the 21 studies directly assessed participants’ expectations, all in the field of pain. Participants rated their expectation regarding impending pain using different scales. Two studies used a visual analog scale (VAS), which ranged from o to 100 (Raghuraman et al., 2019; Schenk and Colloca, 2020). Another study employed a 6-point numeric rating scale (Schwartz et al., 2022a), while the fourth study used an 11-point scale (Vögtle et al., 2019). Two studies assessed expectations right after the observation phase (Raghuraman et al., 2019; Schenk and Colloca, 2020) and found that participants in the OL placebo condition expected less pain compared to participants in the control condition. However, details regarding the specific relation between expectations and the (pain) outcome were not reported. In another study (Vögtle et al., 2019), participants recalled their expectations regarding the effect of a sham ointment retrospectively, that is after they had completed the pain procedure. Participants expected more unfavorable effects in the nocebo condition compared to the control condition, but this difference in expectation was not associated with differences in the actual treatment effects (Vögtle et al., 2019). Thus, expectations did not show a clear association with the outcome. Expectation in these experimental studies was investigated as predictor of the placebo or nocebo response to an upcoming pain stimulus. In contrast, the dynamics of expectation and whether they changed through the intervention was captured in a clinical study with chronic back pain patients that included an assessment of participants’ expectations on pain relief at three time points, that is at baseline before the experiment, postintervention, and posttest (2 week after the experiment) (Schwartz et al., 2022a). While no changes in treatment expectation occurred immediately after the intervention, despite observing an effect on the outcome of pain reduction, there were significant changes 2 weeks later at the posttest, after participants had been interviewed by a physician. According to the authors, the interview might have contributed to participants’ recollection of the positive effects of the medication, thus augmenting the placebo effect. Although not specifically addressed by assessing participants’ expectations, the change of expectations through an intervention has also been considered in a study that traced observationally induced symptom development with a follow-up study (Faasse et al., 2018). Here, results showed that symptoms significantly generalized from specifically modeled symptoms (e.g., headache and dizziness) to a broader range of other, non-modeled symptoms after 24 h. This result was interpreted to mean that negative treatment expectations may generalize to other outcomes over time.

##### 3.1.1.6 Risk of bias and quality assessment

The risk of reporting bias was low throughout all included studies. External validity was only given in the clinical study but not in experimental settings. Internal validity gave rise to concerns in studies in which the blinding of experimenters and/or participants could not be controlled. Due to small sample sizes, some studies lack power and reported effects should be treated with caution. As subjective assessments (pain or side effects) and self-reports (questionnaires) were used in most studies, there is a risk of bias in all studies with regard to blinding of outcome assessment. The results of the bias assessment can be found in the Supplementary Figure S1.

A definitive statement about the certain existence of a placebo or nocebo effect and their effect size is limited in some studies due to the design of the control groups. Although all included studies tested either against a control group (parallel group designs) or included trials with control cues (within-subject-designs), the lack of no-treatment control groups or natural history groups, as well as the fact that in within-subject-designs, participants underwent both, direct conditioning and OL, limits the extent to which the results can be attributed to a placebo or nocebo effect solely. Table S4 summarizes control groups and control conditions. Additionally, two studies reported a lack of control data regarding a specific sub-domain of their respective study (e.g., empathy or social status).

#### 3.1.2 Durability of OL-induced effects

Ten studies investigated temporal profile of OL-induced treatment effects, with eight studies providing insights into the persistence of the elicited effects, and two studies focusing on alterations in treatment expectations over time (refer to section 5.1. for details). Treatment outcomes (usually evaluated with a NRS) were either measured at various post-treatment time points, including follow-ups with a maximum observation period of 2 weeks, or by observing changes during a single experimental run (e.g., by dividing the runs into sub- sets and comparing them, so that changes could be observed from the beginning to the end of the experiment). In a conditioning experiment, extinction trials were used to deliberately weaken or dissolve a previously established association between a conditioned and an unconditioned stimulus, aiming to test whether inhibitory learning of the conditioned cues occurs. Here, extinction trials with non-painful thermal stimuli (34°C) did not lead to the extinction of previously conditioned placebo and nocebo effects (Egorova et al., 2015).

In four studies, stable placebo and nocebo effects were established (Colloca and Benedetti, 2009; Egorova et al., 2015; Raghuraman et al., 2019; Brączyk and Bąbel, 2021).

Two studies reported extinction of placebo analgesia. Bieniek and Bąbel (2021) reported a decrease in overall pain sensation during the experimental session and concluded that this could indicate the extinction of placebo analgesia. Zhang et al. (2017) found a significant difference in the magnitude of the placebo responses between two time points (5-min interval) and that placebo effects significantly decreased over time, while nocebo effects were likely to persist. Nevertheless, the treatment administered on the first day of the experiment was found to influence subsequent treatment outcomes of treatments administered after 5–6 days in both placebo and nocebo conditions (Zhang et al., 2017). Diverging results related to the respective presentation mode were found by Hunter et al. (2014), who report stable effects in the OLv but a trend to extinction in OLp. Buglewicz-Przewoźnik et al. (2022) integrated experimental groups that differed in time between observation and stimulus presentation (several minutes) to test the influence of time on observationally induced effects but found no significant differences between the groups. Moreover, they found that observation prevented habituation to repeated stimulus presentations. Despite some conflicting results and the lack of a standardized method for assessing the temporal dynamics, there is some support to the notion that OL can induce durable effects, particularly for nocebo effects. However, since the maximum observation period was 2 weeks, it remains unclear how OL induced effects develop over longer time periods.

### 3.2 Search 2

**Figure 4:**
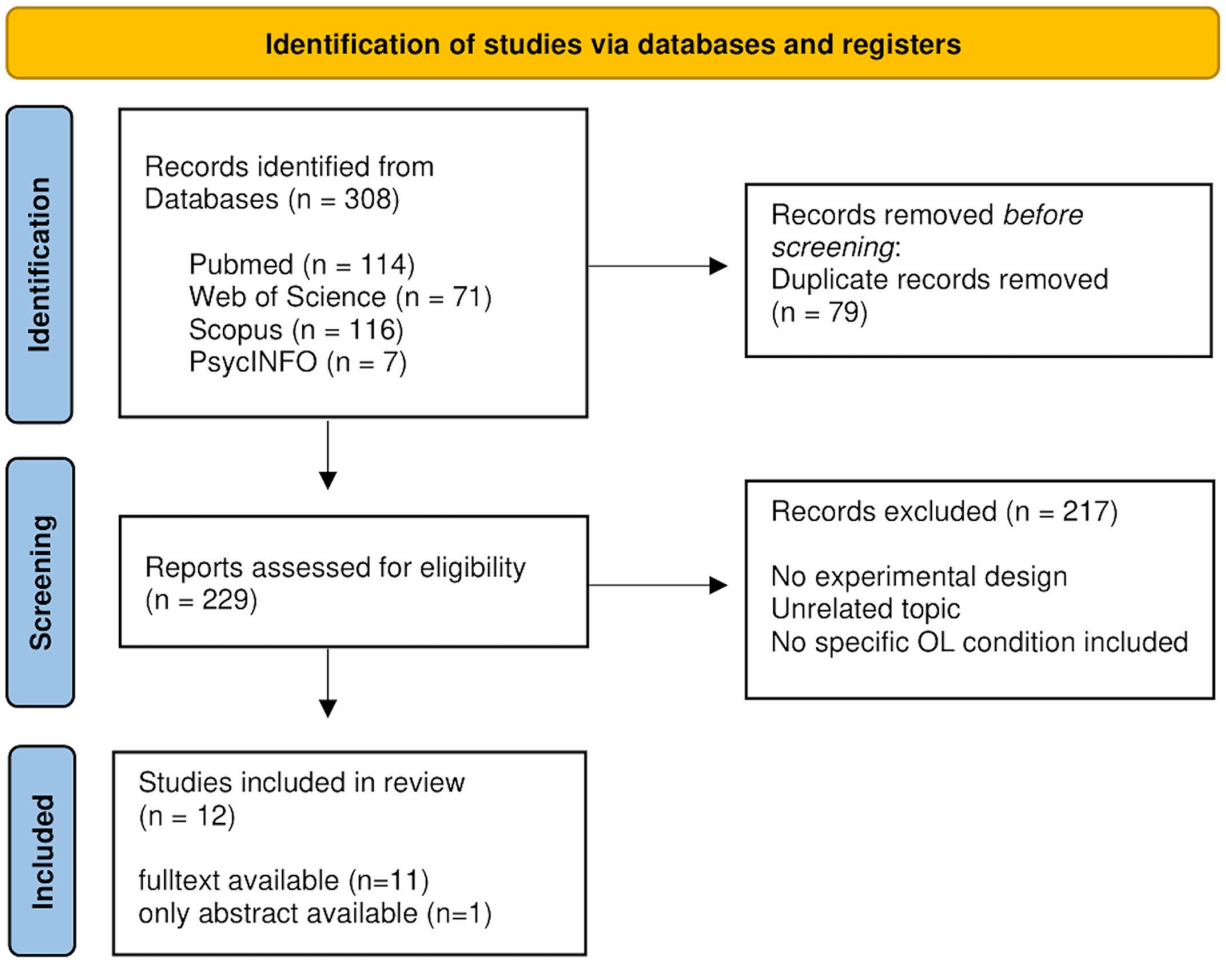
PRISMA flow diagram for data selection process modified from Page et al. (2021) for the second search. The number of initially 308 identified articles was reduced to 12 eligible studies that were included in this review.

For Search 2, we included 12 studies examining OL-induced placebo effects in various medical settings. Most of these studies (*n* = 9) employed a design in which participants in the experimental group were exposed to videos demonstrating a specific behavior. These videos were presented as short video clips (e.g., as DVD or integrated into a mobile phone application) and could be viewed consecutively over several days to months. Only three studies used OLp. We categorized the studies according to their investigation objective, i.e., prevention, treatment or treatment adherence, diagnosis, and rehabilitation. Most interventions were conducted with children or young adults, although some studies also included older patients. Figure 4 illustrates the PRISMA selection process for search 2. For a comprehensive overview of the corresponding research studies, please refer to Supplementary Table S2.

#### 3.2.1 Areas of medical application

**Figure 5:**
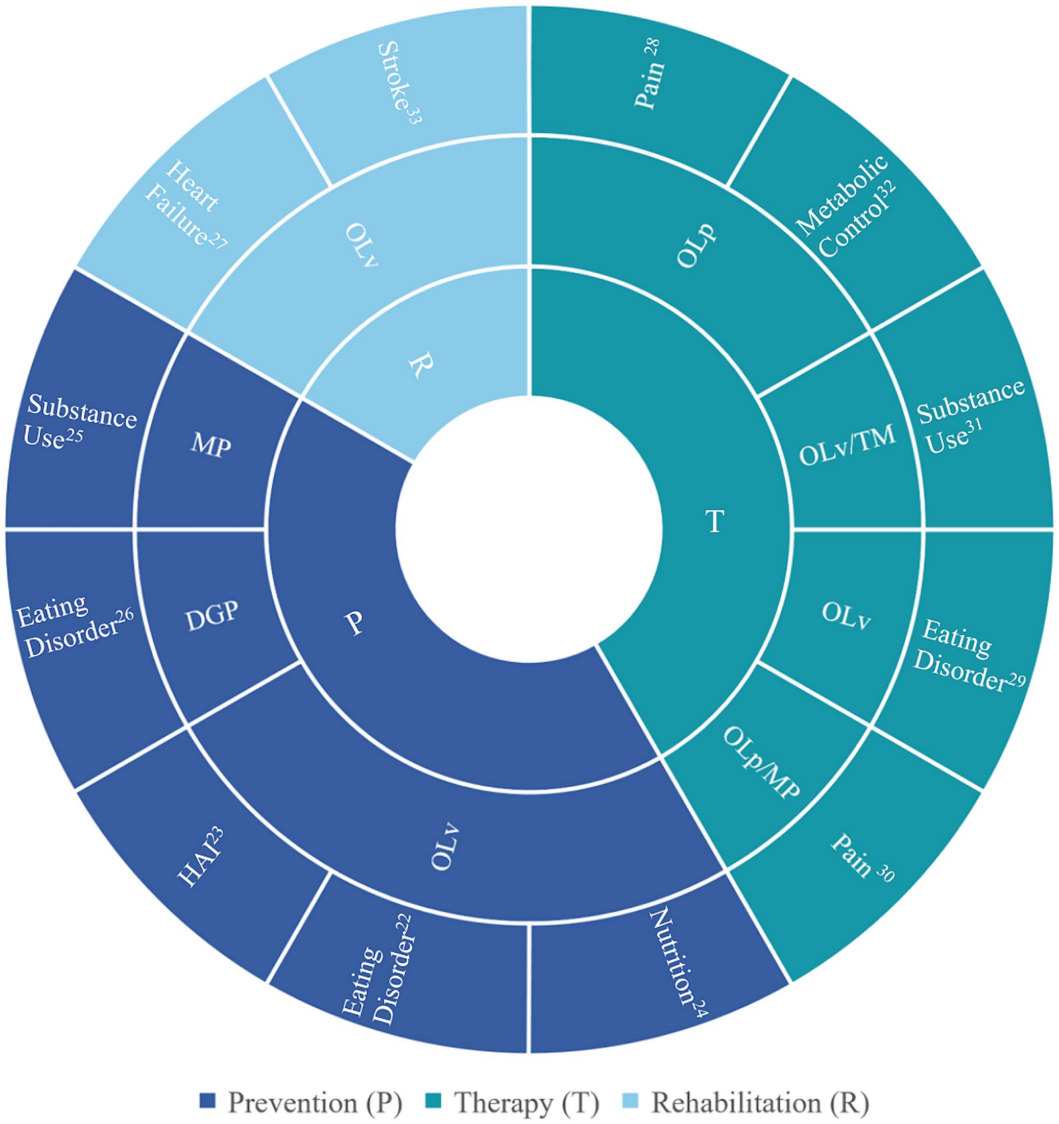
Results of search 2 (*n* = 12) sorted into categories by medical setting (inner circle), observation mode (middle circle), and medical condition (outer circle). For all references, refer to Supplementary Table S2. P, prevention; T, therapy; D, diagnostics; R, rehabilitation; OL, observational learning; OLp, OL in person; OLv, OL via video; TM, text messages; MP, mobile phone; DGP, digital gamified program; HAI, hospital acquired infections (nosocomial infections).

Five of the included studies focused on prevention. These studies examined various health behaviors, including the promotion of healthy eating behavior in early childhood, the encouragement of healthy nutrition in infants, the prevention of eating disorders, the reduction of nosocomial infections in an intensive care unit, and the prevention of substance use. Additionally, five studies targeted therapy. The conditions addressed in these studies were chronic low back pain, obesity, functional abdominal pain, nicotine addiction, and therapy adherence in metabolic control of diabetes. Two interventions aimed at rehabilitation, in patients with heart failure and post- stroke patients, respectively. Figure 5 provides a graphic representation of the results categorized by aspect of medical setting, observation mode, and addressed medical condition.

#### 3.2.2 Observation mode

The studies examined various observation modes such as live, video-based, mobile phone-based, and digitally gamified online OL interventions. Nine studies used OLv, and two studies employed OLp. One study tested OLp and OL by phone (Levy et al., 2017).

#### 3.2.3 Incorporating interventions

Three studies involved active user involvement. In a study concerning diabetes type 1, the participants themselves were responsible for developing a video as part of the intervention (Massouh et al., 1989). In another study, participants guided a fictional character through a gamified intervention (Karekla et al., 2022) and in a third study, participants in a substance use prevention program had the option to select content that interested them and respond to questions via a mobile phone application (Haug et al., 2021). Although the video development intervention by Massouh et al. (1989) did not yield a significant effect, the two more recent digitally delivered interventions both reported significant results, at least for some of the main outcomes.

#### 3.2.4 Magnitude of the effects

The comparison of different medical settings (prevention, therapy, diagnostics, rehabilitation) revealed the largest effects for preventive interventions. This applied especially to a digital gamified body acceptance and commitment early-intervention program for young women at high risk of developing an eating disorder (Karekla et al., 2022). In this game-like program, users accompanied a fictional young girl on her way through decisions related to a fashion contest and observed the story in a third person perspective. However, in comparison to other OLv interventions, users could assist the fictional character and thus participate actively. The intervention resulted in significantly lower weight concerns in participants, compared to a control group (Karekla et al., 2022).

OL showed little to no effect in four therapeutic interventions targeting obesity, metabolic control of insulin-dependent diabetes mellitus, and nicotine addiction but a medium sized effect (*d* = 0.63) in an intervention targeting back pain. Here, functional capacity in low back pain patients improved significantly after the live observation of a model reporting and showing beneficial treatment effects (Schwartz et al., 2022a). Another effective therapeutic intervention targeted functional abdominal pain in children. Here, the OL intervention did not reduce the gastrointestinal symptoms in children but led to significantly greater improvement of parent responses such as solicitousness and pain beliefs. This resulted in fewer healthcare visits for abdominal pain and, in the remote condition, fewer missed school days (Levy et al., 2017). One study focused on rehabilitation aspects in post-stroke patients but found no significant differences in mobility, self-care and usual activities between the intervention and the control groups. Table 3 provides an overview of the study categories and the intervention types from search 2.

**Table 3:** Intervention with highest OL induced effects (search 2).

Study category	Number of studies in each category	Intervention with highest OL induced effect
Prevention	5	Digital gamified intervention with active participation of users
Therapy	5	Live report and demonstration of beneficial effects
Rehabilitation	2	Modeling video (DVD)

Study categories and intervention type of search 2 that achieved the highest effect within the respective category.

#### 3.2.5 Risk of bias and quality assessment

The risk of reporting bias was low in OL application studies. However, the risk of internal and especially external validity was rated as moderate in most of the included studies. Only four studies reported a power calculation, and the sample sizes were generally small. The results of the bias assessment can be found in the Supplementary Figure S3.

## 4 Discussion

This systematic review summarizes our current knowledge about observationally induced placebo and nocebo effects and provides an overview over the medical areas in which OL has been successfully applied to optimize treatment outcomes. Our review expands upon previous systematic reviews of OL- induced placebo and nocebo effects on pain (Schwartz et al., 2022b; Blythe et al., 2023; Meeuwis et al., 2023) by extending the search beyond the field of pain, to nocebo effects, and by adding a perspective on the range of clinical applications of OL-based placebo interventions (search 2). Through outlining remaining gaps of knowledge and important areas of future investigation we thus hope to contribute to a further optimization of OL applications in clinical practice.

### 4.1 The magnitude of OL induced placebo and nocebo effects

Findings from search 1 indicated on average moderate OL effects for each placebo and nocebo effects, encompassing a spectrum of effect sizes from small to large. This implies that OL produces effect sizes comparable to those of first-hand conditioning and surpassing those of verbal suggestion. Nevertheless, different placebo and nocebo induction methods will still need to be compared more directly in future studies to validate this hypothesis. Furthermore, the results showed higher mean values for placebo effects (*d* = 0.79) compared to nocebo effects (*d* = 0.61). However, since this was merely a descriptive difference it remains to be seen whether OL is truly more effective for placebo than for nocebo effects. Apart from the magnitude of measured effects, the durability of the effect is an important aspect regarding the clinical translation of OL effects. However, this can only be assessed to a limited extent, as follow-up studies were only rarely included, and the maximum follow-up period was 2 weeks.

### 4.2 OL in various medical fields

Most of the included studies in search 1 focused on how OL shapes pain perception. However, some other studies showed promising indications that these effects may also exist in other domains, such as shaping side effects or enhancing the effectiveness of medication (e.g., beta-blockers) in general. For clinical studies (search 2), we observed a notable emphasis on mental health and psychosomatic conditions. The focus on nutrition and eating disorders in one-third of these studies may be attributed to the ease of visually presenting eating-related phenomena, making them suitable for OL study designs. However, the interventions and study designs varied greatly, making comparisons and definitive conclusions challenging. The results demonstrated a successful integration of OL into prevention and treatment programs, with prevention interventions achieving larger effects.

### 4.3 Presentation mode

Since most of the included studies used OLv, this systematic review provides its most comprehensive conclusions about video-induced effects. Results of search 1 indicate that OL can successfully induce placebo effects of similar magnitude regardless of the presentation mode (OLv or OLp) (Faasse et al., 2018), an important result with respect to the scalability of interventions. However, a direct comparison of both studies revealed stronger effects for OLp than OLv in one study (Hunter et al., 2014; Meeuwis et al., 2023).

Search 2 showed that the presentation mode for an intervention may depend on the specific medical setting (e.g., prevention, therapy) and should consider practical aspects such as feasibility and adaptability. Interestingly, all preventive interventions were delivered digitally and consistently yielded significant effects. Within a therapeutical setting, an OLp intervention evoked the strongest effect. Regarding prevention, it is likely that OLv (rather than OLp) was chosen and proven to be suitable due to the inherently limited opportunities for face-to-face encounters in medical contexts among healthy participants targeted by prevention interventions. Apps and videos offer convenient means to reach groups for preventive measures and allow for continuous usage and on-demand support. This aligns with the broader trend towards an increased use of digitalized health support through authorized digital health applications. Currently, these apps are predominantly used for mental health conditions (Gerlinger et al., 2021). However, exploring the implementation of OLv in apps to improve other medical conditions, such as reducing side effects or improving therapy adherence, holds promise. Considering that active participation in a gamified intervention (Karekla et al., 2022) yielded the highest effects among all RCTs supports the idea that positive, health-related changes can be achieved through video games and stories. The engaging properties of interactivity, attention-maintenance, and entertainment could contribute to the magnitude of these effects (Baranowski et al., 2008). Future experiments should systematically investigate the correlation between active participation, user involvement, and the magnitude of OL-induced effects. Understanding the underlying mechanisms, such as, e.g., character identification, would improve the development and efficacy of targeted health applications as they can achieve high levels of standardization while concurrently catering to individual needs, e.g., through personalized presentations and AI implementations.

### 4.4 Sex/gender

The sex/gender of models and observers is a widely studied variable in OL experiments as well as in pain studies. A previous systematic review found that sex differences in placebo and nocebo effects exist, and that women and men respond differently to different experimental methods (verbal information and conditioning) (Vambheim and Flaten, 2017). Moreover, a study on placebo analgesia showed sex differences in placebo analgesia, when the placebo effect could be enhanced by vasopressin in women but not in men (Colloca et al., 2016). Considering that this systematic review mainly included pain-related studies, and that differences in sex seem to be influenced by the experimental method, these earlier results suggested that this review could show clear differences in OL-induced placebo and nocebo effects between women and men. However, although some evidence suggests that OL elicits stronger responses in female observers, not all studies report significant gender differences, and the influence of gender on the magnitude of OL-induced effect sizes remains inconclusive. It is worth noting that eight studies included only female participants, while no study exclusively included males. This may be due to earlier reports that observing a model lead to greater nocebo hyperalgesia (Swider and Babel, 2013) and symptom increase (Lorber et al., 2007) in female observers as compared to men. As the evidence regarding gender effects in OL remains relatively limited, with a primary focus on nocebo effects and adverse outcomes, we believe that a more systematic and balanced investigation of gender-related effects would be valuable. This pertains not only to the investigation of OL-induced treatment effects but also to the exploration of sex and gender differences in placebo and nocebo effects in a broader context (Shafir et al., 2022). Moreover, there is a notable discrepancy in sex differences concerning placebo and nocebo effects among different study types, with evidence of such differences being more prominent in experimental studies compared to RCTs (Enck and Klosterhalfen, 2019).

### 4.5 Empathy

In five studies, empathy was associated with or even predictive of the magnitude of placebo/nocebo effects, while nine studies reported no significant association. These findings suggest that empathy, particularly empathic concern, may influence OL-induced placebo and nocebo effects, but that this influence may vary, depending on the observation mode (OLp vs. OLv) (Hunter et al., 2014). Recent research on social transmission of symptoms also supports the notion that trait empathy may be less involved in OLv (Tan et al., 2023). High empathy scores thus do not appear to be a general prerequisite for eliciting OL effects, and some studies outside of placebo/nocebo research report that higher trait empathy may even have a detrimental effect on OL (Kobza et al., 2011; Rak et al., 2013). On the one hand, the variability in results can be attributed to the intricate role of empathy in OL. On the other hand, it highlights the challenge of precisely defining and measuring empathy in research. Thus, whether empathy plays a measurable role may depend on the specific measurement and aspect of empathy being considered. In this sample, all included studies used the Interpersonal Reactivity Index (IRI) to assess empathy as a disposition or personality trait. However, there are important considerations. Firstly, it is possible that situational empathy may be more relevant than trait empathy in the context of OL. Secondly, Baron-Cohen and Wheelwright (2004) pointed out that some established instruments for measuring empathy, including the IRI, do not focus exclusively on empathy but also include broader aspects (e.g., “I dream and fantasize with some regularity about things that might happen to me”). Furthermore, studies investigating the observational acquisition of threat responses have indicated that empathy traits [measured here with the Balanced Emotional Empathy Scale (BEES, Mehrabian, 1996)] might have some impact on OL, but only when participants are explicitly instructed to empathize (Olsson et al., 2016), although see Williams and Conway (2020) for differing findings]. This raises the question of whether the currently established measurement tools adequately capture the multifaceted role of empathy in OL placebo/nocebo research.

In conclusion, the varying results regarding empathy’s role in OL investigations may be attributed to differences in how empathy is assessed, or it could imply that empathy might be less influential in OL, and further research is needed to clarify this matter.

### 4.6 Other observer and model characteristics

Anxiety, fear of pain, individual pain sensitivity, and social conformity did not show significant correlations with placebo or nocebo effects. While one study reported a significant correlation with pain catastrophizing (specifically the subscale helplessness), this finding was not specific to the OL condition and could not be replicated in subsequent studies.

When examining model characteristics such as social status and self-esteem, placebo effects were observed in the experimental groups, where participants watched a video, in contrast to the respective control groups, where no video was shown. However, when comparing different degrees of expression of the examined characteristics (e.g., low status and high status) within different experimental groups, no significant differences in effect size were found. This suggests that the placebo or nocebo effects elicited are primarily modulated by OL and are less dependent on the varying degrees of expression of the investigated model characteristics. Consequently, our understanding of the relevant observer and model characteristics remains incomplete. Previous studies have highlighted the importance of characteristics such as trustworthiness, expertise (Lirgg and Feltz, 1991; Rakoczy et al., 2009), and attractiveness of a model (Makuch et al., 2011). Trustworthiness assessments have been associated with facial cues (Sofer et al., 2015; Kaisler and Leder, 2016), while attractiveness influences how observers perceive a model’s social status (Little et al., 2011), which has already been linked to OL- induced placebo analgesia (Bieniek and Bąbel, 2021). However, the exact facial characteristics that mediate the effect of faces as stronger cues for pain conditioning (Egorova et al., 2017) are yet to be clarified. To further elucidate the role of model-observer relationship in terms of similarity and familiarity in OL, experiments should consider age, appearance, and other shared characteristics such as suffering from the same disease. In addition, other variables that produce a subjective sense of closeness, such as sympathy, shared values, and humor, could be explored.

### 4.7 Expectation

Treatment expectation as an inherent component of placebo and nocebo effects was of particular interest in this review. However, individual expectations were only directly assessed in a few studies, and there were only partial correlations between expectations and treatment outcomes reported. This result highlights the difficulty of precisely defining the construct of expectation in the treatment context on the one hand, and of surveying it accordingly on the other. Distinguishing between different forms of expectation, such as implicit, explicit, and generalized expectations and meta-cognitions, e.g., self- efficacy and optimism (Laferton et al., 2017), may be important. The common understanding of expectations being explicit and conscious (Bajcar and Bąbel, 2018) is challenged by the fact that (observational) conditioning can bypass explicit expectations and still induce measureable placebo and nocebo effects (Egorova et al., 2015; Bräscher et al., 2017, 2018). Furthermore, research on related fields, e.g., the observational acquisition of threats provided evidence for implicit routes (Olsson and Phelps, 2004) and thus supports the hypothesis of OL-induced effects through implicit expectations. However, to date, the roles of explicit and implicit expectation in OL-induced placebo and nocebo effects have not been sufficiently investigated and warrant further investigation. Specific research on this may be of particular interest and relevance to clinical populations, such as patients with Alzheimer’s disease, whose formation of explicit expectations and cognitively triggered symptom modulation, such as pain modulation, may be impaired (Benedetti et al., 2006; Matthiesen et al., 2019; James et al., 2021).

Another key challenge in assessing the role of (treatment) expectation in OL-induced effects, as well as in placebo effects more broadly, is the lack of a commonly used tool to measure expectations in treatment contexts. Recent developments such as the Generic rating scale for previous treatment experiences, treatment expectations, and treatment effects (GEEE) (Rief et al., 2021), or the Treatment Expectation Questionnaire (TEX-Q) (Shedden-Mora et al., 2023) represent important steps towards this goal.

### 4.8 OL influencing factors in RCTs

In search 1, our focus was on factors influencing OL and the circumstances that might facilitate it, while Search 2 provided insights into the use of OL in clinical trials. By synthesizing the findings from both searches, we gain a comprehensive understanding of how experimentally derived findings may be translated into clinical applications. One intriguing observation is that efforts to implement OL in clinical proof-of-concept studies began as early as the late 1970s, long before the first experimental study was conducted in 2009. This suggests that these two research directions - experimental and applied – seem to have developed somewhat independently of each other. This could potentially explain the difference in the medical application fields. While the RCTs on OL were primarily, though not exclusively, focused on mental illnesses (e.g., addiction and eating disorders), the experimental studies clearly focused on the investigation of pain perception. However, some commonalities between the two approaches can still be identified, and the goal of this work is also to reconcile these different approaches. One notable commonality is the investigation of the relationship between the observer and the model, an aspect that was approached in various ways.

In search 1, we found that similarities between the model and observer in terms of gender and age were taken into account. In the studies from search 2, models were predominantly selected according to the target population of the respective intervention. For example, actors in videos represented parents when the intervention targeted parents (Black and Teti, 1997; Ledoux et al., 2018), and models were patients when the intervention was aimed at patients (Maddison et al., 2008) or caregivers (Jones et al., 2018). In one study on type 1 diabetes, videos were even developed and shot involving the affected individuals themselves (Massouh et al., 1989), creating maximum congruence between model and observer.

Similarly, the gender of a model appears to have been considered when the intervention under study was directed at a particular gender group. For instance, in a study with females at high risk for eating disorders, a fictional female character was used as a model (Karekla et al., 2022).

Ethnicity and cultural background were also taken into account in several studies. In a smoking cessation study conducted by the University of Auckland, participants were given choices (Whittaker et al., 2011), or models were selected to match the ethnicity of study participants (Black and Teti, 1997; Ledoux et al., 2018). This was done possibly to create a sense of similarity and relatability between participants and models. However, other person-related variables that have been investigated in experimental studies, such as status or empathy, seem to have played a lesser role in the design of RCTs. This suggests that there may be room for further exploration and integration of these variables into the design of clinical trials involving OL interventions.

### 4.9 Considerations for clinical practice and outlook

One of the aims of this systematic review was to infer recommendations for the targeted implementation of OL in medical practice. However, to date the existing studies do not provide enough evidence to develop clear guidelines. Nonetheless, we can draw several interim conclusions that may inform future research and hold clinical implications. Overall, we found that in medical settings OL can help alleviate symptoms and can be effectively used in preventive, therapeutic, and rehabilitative interventions, with highest effects for preventive interventions. Most studies focused on conditions that are strongly influenced by subjective perception and involve psychosomatic components and interoceptive processing. The results further suggest that embedding a video into a gamified digital health application might be the most effective approach for OLv. In either case, the observer’s attention should be carefully controlled in studies using observation. Findings revealed that conscious stimulus perception leads to greater effects than subliminally presented stimuli (Egorova et al., 2015; Tu et al., 2019). For completeness, it should be noted that previous research has shown that only one person rather than a group should serve as a model (Bajcar et al., 2022) and that facial expressions rather than other types of cues elicit higher effects (Egorova et al., 2017).

While the magnitude of OL effects was consistently reported and showed that OL can evoke significant effects, the sustainability of these effects is still poorly understood. To facilitate the translation of experimentally tested effects into practice both effect sizes and the persistence of effects are highly relevant. Future studies should thus investigate the temporal dynamics of OL-induced effects over more extended periods of time.

It is also important to note that OL effects are not restricted to positive treatment outcomes (placebo effects). OL can induce substantial nocebo effects, which can persist over time. This underscores the importance of careful communication between health care providers and patients, and among patients. One implication of this would be to shield patients from each other in critical situations to prevent OL- induced nocebo effects. This applies particularly to patients who suffer from a similar condition or share several commonalities, as they might relate more strongly to their fellow patients’ suffering. Regarding the use of digital health applications, the optimal balance between physical patient – provider interactions needs to be found, and patients’ preferences should be taken into consideration.

Taken together, the current literature shows that OL can be used effectively to modulate treatment outcomes in various medical domains with similar effectiveness of OLv and OLp. Concrete guidelines for practice cannot be derived from the results yet, as many findings are still contradictory and important comparisons of conditions are lacking. We highlight important areas for future research, such as the role of sex/gender differences, empathy, and attention/engagement with the interventions. OL may hold particularly great promise with respect to (potentially AI-supported) digital health interventions. Context- and application specific implementation of influencing variables, optimal “dosing” and timing of OL interventions along patients’ treatment trajectories as well as longer-term treatment effects of OL should also be investigated in future studies.

## 5 Limitations

Despite a relatively small number of hits, the broad systematic search resulted in a very heterogeneous data set, so that although the data were compared descriptively, a meta-analysis of the results and definite comparison of effect sizes was not possible. Although we converted all units of effect sizes to Cohen’s d when otherwise specified to facilitate comparability, the heterogeneity of study designs and the influencing factors investigated limits the comparability of effect sizes between studies. This heterogeneity of the studies also limits the comparability and thus the reliability with regard to the certainty with which an overall effect can be attributed to a particular influencing factor. The evidence regarding the size of the placebo and nocebo effects described is limited, as some of the included studies did not include a no-treatment control group in the study design. As the terms “sex” and “gender” are not meaningfully differentiated in the primary literature, this study does not provide any information on possible differences in OL-induced effects between these gender terms. We did not analyze the relationship between participants and models to the respective study experimenters. In the studies, different numeric scales were being used to assess pain. We did not look into potential anchoring bias effects that might have had arised when participants observed pain ratings previously given by a model. Thus, a potential contribution of anchoring bias in reported OL effects cannot be ruled out. A further limitation is that the results of studies with patients and healthy participants are only comparable to a limited extent (Forsberg et al., 2017). Although identified by the databases, three studies did not fulfill all criteria for RCTs, one of which could not be fully evaluated (please see Supplementary Table S2).

### Registration and protocol

This work was preregistered at the Center for Open Science as open-ended registration (doi: 10.17605/OSF.IO/FVHKE). The protocol can be found here: https://archive.org/details/osf-registrations-fvhke-v1.

## Data availability statement

The original contributions presented in the study are included in the article/Supplementary material, further inquiries can be directed to the corresponding authors.

## Author contributions

HK: Conceptualization, Investigation, Writing – original draft. AK: Conceptualization, Supervision, Writing – original draft, Writing – review & editing. DM: Writing – review & editing. JH: Writing – review & editing. UB: Conceptualization, Funding acquisition, Supervision, Writing – review & editing.
