# Supplementary material for: Learning by observing: a systematic exploration of modulatory factors and the impact of observationally induced placebo and nocebo effects on treatment outcomes
Source: Front Psychol. 2024 Apr 18;15:1293975. doi: 10.3389/fpsyg.2024.1293975 (PMC11064928; doi:10.3389/fpsyg.2024.1293975)
Supplement: Supplementary file 1 [file Data_Sheet_1.PDF]

Supplementary Material

Learning by observing: A systematic exploration of modulatory factors and the impact of observationally induced placebo and nocebo effects on treatment outcomes

Helena Klauß[1], Angelika Kunkel[1*], Diana Müßgens[1], Jan Haaker[2], Ulrike Bingel[1]

[1]Department of Neurology, Center for Translational Neuro- and Behavioral Sciences (C-TNBS), University Medicine Essen, University Duisburg-Essen, Hufelandstr. 55, 45147 Essen, Germany

[2]Department of Systems Neuroscience, University Medical Center Hamburg-Eppendorf Martinistr. 52. 20246 Hamburg, Germany

*** Correspondence:**
Angelika Kunkel
Angelika.Kunkel@uk-essen.de

1 Supplementary Tables

Table S1

Search Queries

Data Base	First Search	Second Search
PubMed	("placebo effect"[Title/Abstract] OR "placebo response"[Title/Abstract] OR "nocebo effect"[Title/Abstract] OR "nocebo response"[Title/Abstract]) AND ("Observational Learning"[Title/Abstract] OR "Social Learning"[Title/Abstract] OR "Social Modeling"[Title/Abstract] OR "Social Observation"[Title/Abstract] OR "Socially induced"[Title/Abstract] OR "Observationally induced"[Title/Abstract] OR "Vicarious Learning"[Title/Abstract])	((intervention[Title/Abstract] OR treatment[Title/Abstract] OR therapy[Title/Abstract] OR surgery[Title/Abstract]) AND (Observational learning[Title/Abstract] OR social learning[Title/Abstract] OR social modeling[Title/Abstract] OR social observation[Title/Abstract] OR socially induced [Title/Abstract] OR observationally induced [Title/Abstract] OR vicarious learning[Title/Abstract)) and Filter: Randomized Controlled Trial
Web of Science	TS= ("placebo effect" OR "placebo response" OR "nocebo effect" OR "nocebo response") AND TS=("observational learning" OR "social learning" OR "social modeling" OR "social observation" OR "socially induced" OR "observationally induced" OR "vicarious learning")	TS= ("intervention" OR "treatment" OR "therapy" OR "surgery") AND TS=("observational learning" OR "social learning" OR "social modeling" OR "social observation" OR "socially induced" OR "observationally induced" OR "vicarious learning") AND ALL=("randomized controlled trial")
Scopus	(TITLE-ABS-KEY (*"placebo effect"*) OR TITLE-ABS-KEY (*"placebo response"*) OR TITLE-ABS-KEY (*"nocebo effect"*) OR TITLE-ABS-KEY (*"nocebo response"*)) AND (TITLE-ABS-KEY (*"observational learning"*) OR TITLE-ABS-	(TITLE-ABS-KEY (*"intervention"*) OR TITLE-ABS-KEY (*"treatment"*) OR TITLE-ABS-KEY (*"therapy"*) OR TITLE-ABS-KEY (*"surgery"*)) AND (TITLE-ABS-KEY (*"observational learning"*) OR TITLE-ABS-KEY (*"social learning"*) OR TITLE-

	KEY (*"social learning"*) OR TITLE-ABS-KEY (*"social modeling"*) OR TITLE-ABS-KEY (*"social observation"*) OR TITLE-ABS-KEY (*"socially induced"*) OR TITLE-ABS-KEY (*"observationally induced"*) OR TITLE-ABS-KEY (*vicarious* AND *learning*))	ABS-KEY (*"social modeling"*) OR TITLE-ABS-KEY (*"social observation"*) OR TITLE-ABS-KEY (*"socially induced"*) OR TITLE-ABS-KEY (*"observationally induced"*) OR TITLE-ABS-KEY (*vicarious* AND *learning*)) A ND TITLE-ABS (*"randomized controlled trial"*)
PsycINFO	((placebo effect or placebo response or nocebo effect or nocebo response) and (observational learning or social learning or social observation or socially induced or observationally induced or vicarious learning)).mp. [mp=title, abstract, heading word, table of contents, key concepts, original title, tests & measures, mesh word]	((intervention or treatment or therapy or surgery) and (observational learning or social learning or social observation or socially induced or observationally induced or vicarious learning) and (randomized clinical trial)).mp. [mp=title, abstract, heading word, table of contents, key concepts, original title, tests & measures, mesh word]

Note. Lists of the search terms and operators used in the systematic literature search for both searches. First search: placebo and nocebo effects through observational learning (OL). Second search: the application of OL in medicine. Databases: PubMed, Web of science, Scopus, and PsycINFO.

Table S2

Results of the Second Search

Authors and year of publication	Setting (prevention, therapy, diagnostics, rehabilitation)	Addressed medical condition/addressed population	Participants	Presentation mode	Duration of study	Main outcomes
Ledoux et al. (2018)[22 P]	Prevention	Obesity/ Responsive feeding and healthy eating behavior in early childhood	Parents<br><br>n = 50	OLv<br><br>(Duration of video: 20 min)	1 week	Significant difference in knowledge change scores; no significant differences in beliefs about unresponsive practices
Chen and Chiang (2007)[23 #]	Prevention	Nosocomial infections in ICU	Families/ parents<br><br>n = 123	OLv<br><br>(Duration of video: 10 min)	5 weeks	Significant improvement in accuracy and compliance in hand washing skills in both groups; significantly greater accuracy improvement in the experimental group

Black and Teti (1997)[24]	Prevention	Healthy nutrition and responsive parent-infant Communication	First-time, African American adolescent mothers of infants<br><br>n = 59	OLv<br><br>(Duration of video: 15 min)	Baseline +2-3 weeks-follow-up	Significant improvement of attitudes and behavior (altering mealtime attitudes and enhancing maternal mealtime communication)
Haug et al. (2021)[25]	Prevention	Substance use	Secondary students<br><br>n = 1473	Mobile phone-based program including interactive features<br><br>(2-4 messages per week)	22 weeks + 6-and 18-months-follow-ups	Significant effect regarding the quantity of alcohol consumed per month, quantity of cigarettes smoked per month, and reported stress
Karekla et al. (2022)[26]	Prevention	Eating disorders	Young females showing symptoms or being at risk for eating disorders<br><br>N=89	Digital gamified program<br><br>(6 sessions)	*n.g.* + 1-month follow-up	Significantly lower shape and weight concerns
Maddison et al. (2008)[27]	Rehabilitation	Heart failure	Patients with chronic heart failure<br><br>n = 20	OLv<br><br>(Duration of video: 10 min)	1 week	Increase in PVO_2 and self-efficacy

Schwartz et al. (2022)[28]	Therapy	Chronic low back pain	Patients with chronic low back pain<br><br>n = 44	OLp	Baseline<br>+ post-test after 2 weeks	Analgesic placebo effect in both groups; significant augmentation of placebo effect on functional capacity, but not on pain intensity
Perry et al. (1979)[29 --]	Therapy (Modifying selected eating responses)	Obesity	Children<br><br>N=18	OLv<br><br>(4 sessions)	*n.a.*	Significant influence of model treatments on dependent measures of eating behavior.
Levy et al. (2017)[30]	(Cognitive behavioral) Therapy	Functional abdominal pain (FAPD)	Children with FAPD and their parents<br><br>n = 316	OLp or phone<br><br>(3 sessions)	3 weeks<br>+ 3- and 6-months follow-ups	No significant changes in pain severity in children; significantly greater reductions in solicitousness, catastrophizing, and pain beliefs, specifically threat appraisals, and significantly greater increase in child use of emotion- and problem-focused coping, according to parents' reports
Whittaker et al. (2011)[31]	Therapy (Smoking cessation)	Nicotine addiction	Younger adults, current daily smokers	OLv and text messages<br><br>(automated and on demand)	weeks<br>+ 6-month follow-up	*n.s.*

			ready to quit smoking<br><br>n = 226			
Massouh et al. (1989)[32 #]	Therapy Adherence	Metabolic control of insulin-dependent Diabetes mellitus	Adolescents with Diabetes mellitus Type 1<br><br>n = 30	OLp<br><br>(daily 40 min role model training)	8 days<br>+ 3,5-month follow-up	*n.s.*
Jones et al. (2018)[33]	Rehabilitation	Stroke	n = 54<br><br>Adults up to three years post stroke with disabilities and their nominated informal caregivers	OLv<br><br>(weekly episodes of DVD)	6 weeks<br>+ 2-months follow-up	*n.s.*

Note. List of RCTs on the application of OL in medicine identified during the second search. RCTs are sorted according to their medical setting (prevention, diagnostic, therapy, or rehabilitation). Information about participant characteristics, presentation mode (live or video) and video duration, duration of study, and main study outcomes are summarized.
Abbreviations: OL= observational learning; OLp= observational learning in person (live, face-to-face); OLv= observational learning by video; *n.s.* = not significant*; n.a.*= not accessible; *n.r.*= not reported; [P]= pilot study [#]=criteria for RCTs only partially fulfilled; [--] no fulltext available

Table S3

Adjustments of the Downs and Black (1998) scale for assessment of study quality.

Item number	Item	Reason for exclusion or modification
8	Have all important adverse events that may be a consequence of the intervention been reported?	No information about adverse events were given, however, due to the experimental methods used, adverse events were not likely.
9	Have the characteristics of patients lost to follow-up been described?	Very few studies included a follow up, and if they did, losses were taken into account in the data analyses. However, most studies didn't include a follow up at all.
11	Were the subjects asked to participate in the study representative of the entire population from which they were recruited?	Information about the representativeness of the sample was no accessible.*
12	Were those subjects who were prepared to participate representative of the entire population from which they were recruited?	Information about the representativeness of the sample was no accessible.
13	Were the staff, places, and facilities where the patients were treated, representative of the treatment the majority of patients receive?	Given the fact that the sample of search 1included 20 (overall n=21) experimental studies, external validity was not given due to the nature of these studies.*
16	If any of the results of the study were based on "data dredging", was this made clear?	The results of studies included in out sample were unlikely to be based on data dredging, given the relatively small number of participants and the limited datasets from which the results were obtained (Meeuwis et al., 2023).
22	Were study subjects in different intervention groups or were the cases and controls recruited over the same period?	Although this did not emerge from the studies, it is very likely that this was the case in all studies.
26	Were losses of patients to follow-up taken into account?	Few studies included a follow up, and if they did, losses were taken into account in the data analyses.

27	Did the study have sufficient power to detect a clinically important effect where the probability value for a difference being due to chance is 5%?	We adjusted the scale:<br>0 points: No power calculation or no possibility to determine its existence from the publication<br>1 point: power = 0.8<br>2 points: power > 0.8

Note: The table shows the item number, item and reason of exclusion of the item or its modification. Some items were changed based on suggestions from Meeuwis et al. (2023).
*item changed only for bias assessment of search 1 results but unchanged for assessment of search 2 results.

Table S4

Control groups and control conditions of search 1 studies.

	Study Design			
	Between-subject-design		Within-subject-design	
Control conditions	Control group **without observation, no demonstrator involved** (n = 10) 2,5,6,9,10,11,13,14,15,17,20	Control group **with observation**, demonstrator involved (n = 5) 1,3,4,16,18	Control condition **without observation, no demonstrator involved** (direct conditioning) (n = 2) 7,21	Control condition **with observation,** demonstrator involved (n = 2) 12,19

Note. The table presents either control groups or control conditions depending on the study design. The control groups/conditions included either observation (e.g., a *neutral* stimulus) or implemented trials *without any demonstration* involved. The numbers refer to the respective studies as per Table 1.

2 Supplementary Figures

Figure S1

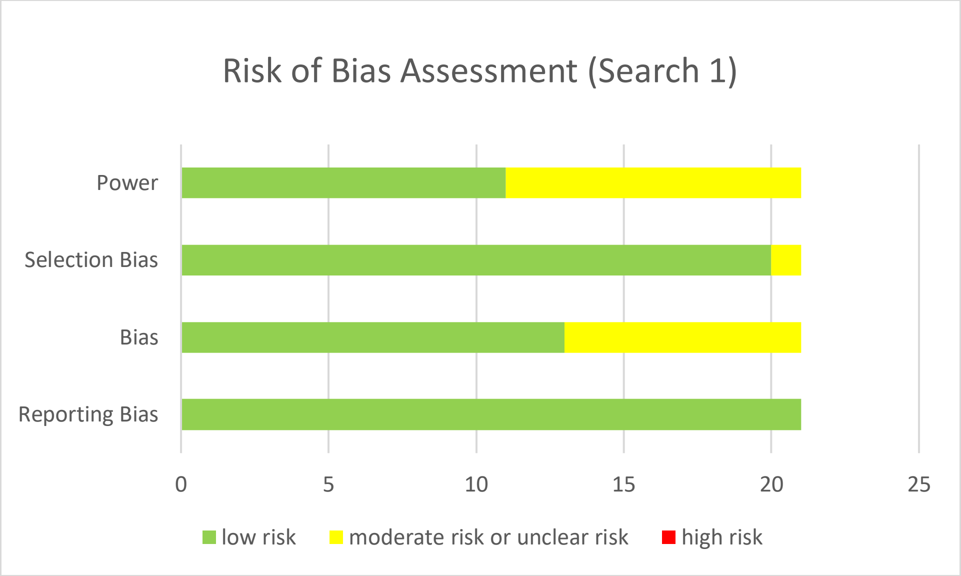

Note. Risk of Bias Assessment.
X-axis: Number of evaluated studies. Y-axis: Type of Bias. Results from the modified Downs & Black checklist were grouped into categories low risk, moderate risk or unclear risk, and high risk (colour code).

Figure S2

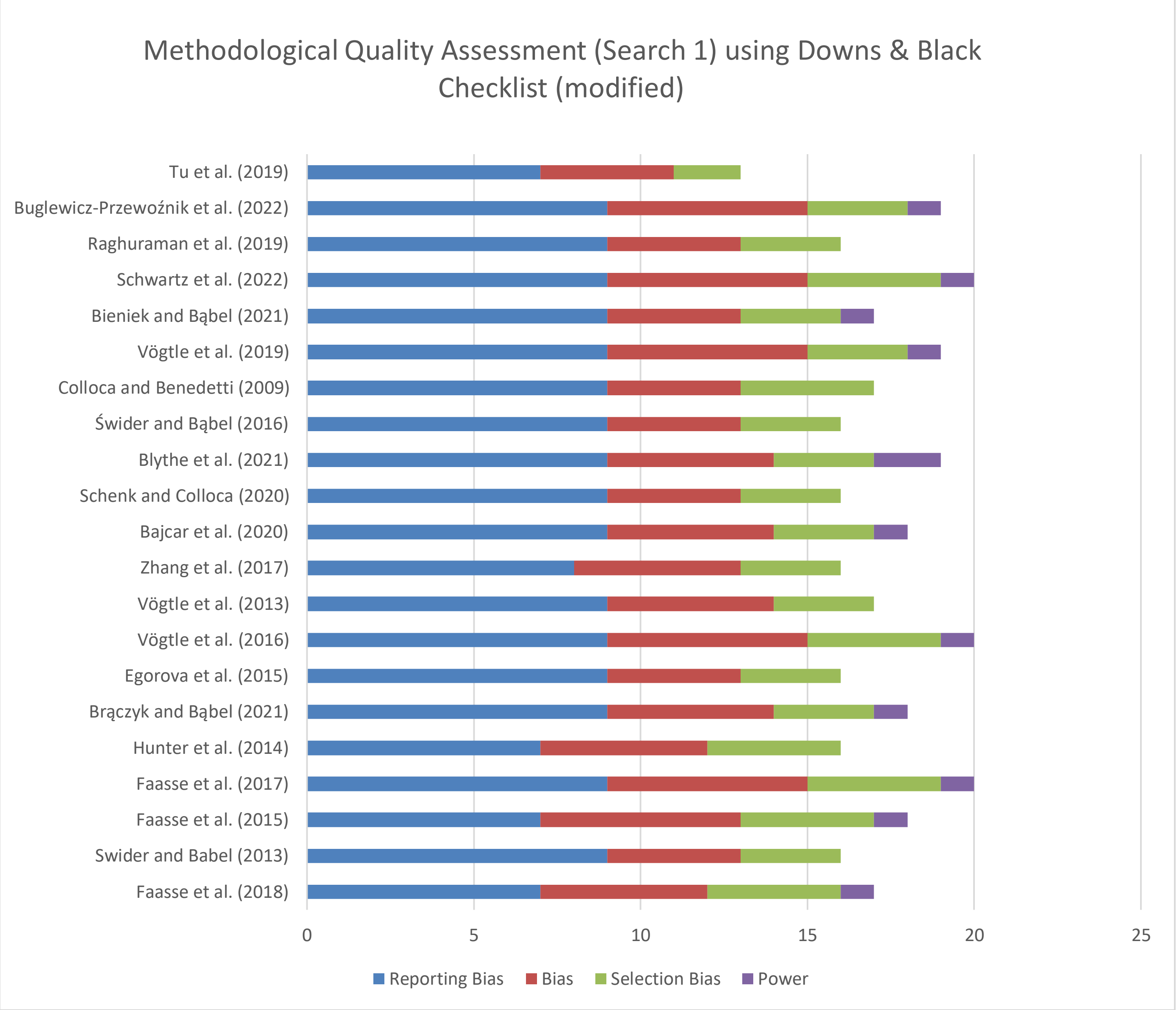

Note. Methodological quality assessment using Downs & Black checklist (modified, see table S4). Quality assessment of the studies regarding reporting, bias, selection bias, and power. After modification of the scale, maximum of points is 21.

Figure S3

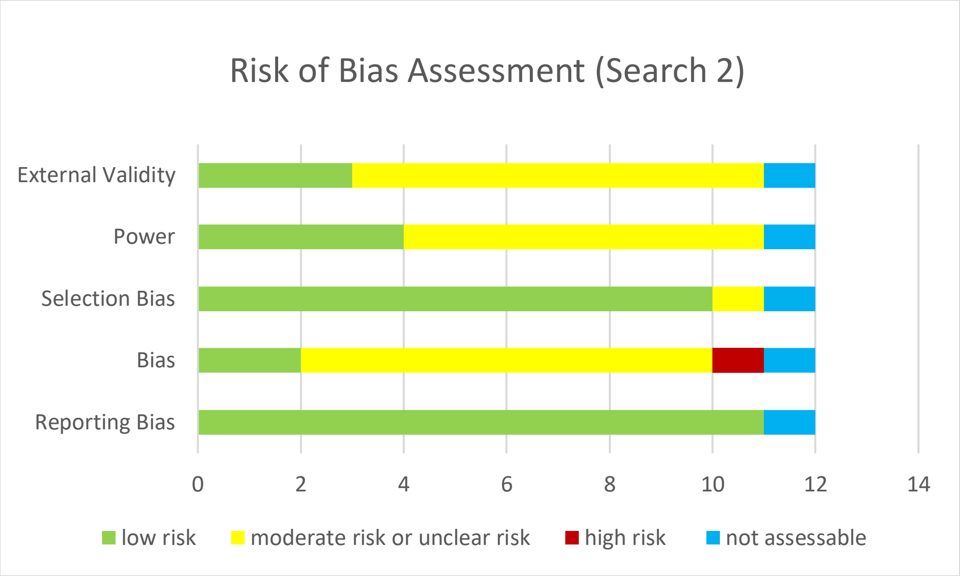

Note. Risk of Bias Assessment.
X-axis: Number of evaluated studies. Y-axis: Type of Bias. Results from the modified Downs & Black checklist were grouped into categories low risk, moderate risk or unclear risk, high risk, and not assessable (colour code).

Figure S4

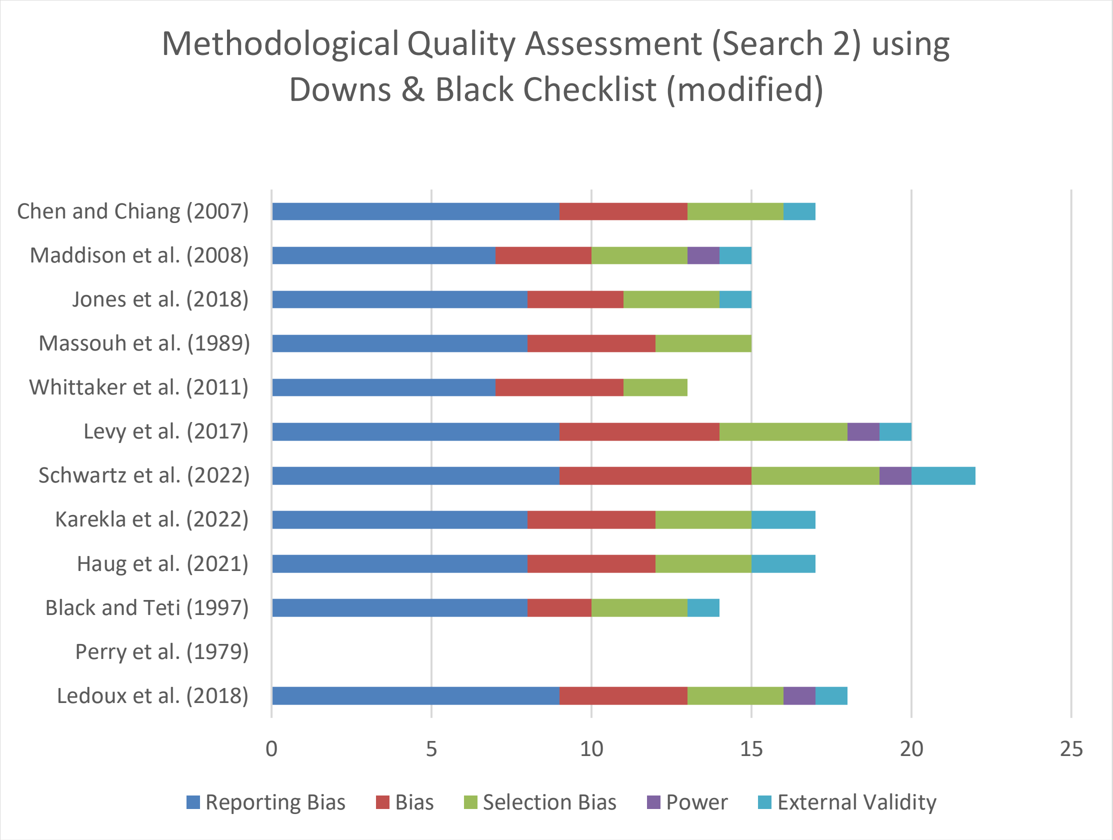

Note. Methodological quality assessment for search 2 using Downs & Black checklist (modified, see table S4). Quality assessment of the studies regarding reporting, bias, selection bias, power, and external validity. After modification of the scale, maximum of points is 23.

3 Supplementary References

Bajcar, E. A., Wiercioch-Kuzianik, K., Farley, D., Adamczyk, W. M., Buglewicz, E., & Bąbel, P. (2020). One of us or one of them? The effects of the model's and observer's characteristics on placebo analgesia induced by observational learning. PLOS ONE, 15, e0243996. doi:10.1371/journal.pone.0243996

Bieniek, H., & Bąbel, P. (2021). The Effect of the Model's Social Status on Placebo Analgesia Induced by Social Observational Learning. Pain Medicine, 23, 81-88. doi:10.1093/pm/pnab299

Black, M. M., & Teti, L. O. (1997). Promoting mealtime communication between adolescent mothers and their infants through videotape. Pediatrics, 99, 432-437. doi:10.1542/peds.99.3.432.

Blythe, J. S., Peereman, K. J., Veldhuizen, D. S., Van Schothorst, M. M. E., Thomaidou, M. A., Van Laarhoven, A. I. M., & Evers, A. W. M. (2021). Nocebo Effects on Cowhage-evoked Itch: A Randomized Controlled Trial of Classical Conditioning and Observational Learning. Acta Derm Venereol, 101, adv00370. doi:10.2340/00015555-3723

Brączyk, J., & Bąbel, P. (2021). The Role of the Observers' Perception of a Model's Self-Confidence in Observationally Induced Placebo Analgesia. J Pain, 22, 1672-1680. doi:10.1016/j.jpain.2021.06.001

Buglewicz-Przewoźnik, E., Adamczyk, W. M., & Bąbel, P. (2022). Is Pain Contagious? Innocuous Stimulation Can be Transformed Into the Pain Experience by Observational Learning. J Pain. doi:10.1016/j.jpain.2022.07.015

Chen, Y. C., & Chiang, L. C. (2007). Effectiveness of hand-washing teaching programs for families of children in paediatric intensive care units. J Clin Nurs, 16, 1173-1179. doi:10.1111/j.1365-2702.2007.01665.x.

Colloca, L., & Benedetti, F. (2009). Placebo analgesia induced by social observational learning. Pain, 144, 28-34. doi:10.1016/j.pain.2009.01.033

Downs, S. H. & Black, N. (1998). The feasibility of creating a checklist for the assessment of the methodological quality both of randomised and non-randomised studies of health care interventions. J Epidemiol Community Health, 52**,** 377-84.

Egorova, N., Park, J., Orr, S. P., Kirsch, I., Gollub, R. L., & Kong, J. (2015). Not seeing or feeling is still believing: conscious and non-conscious pain modulation after direct and observational learning. Scientific Reports, 5, 16809. doi:10.1038/srep16809

Faasse, K., Grey, A., Jordan, R., Garland, S., & Petrie, K. J. (2015). Seeing is believing: Impact of social modeling on placebo and nocebo responding. Health Psychol, 34, 880-5. doi:10.1037/hea0000199

Faasse, K., Perera, A., Loveys, K., Grey, A., & Petrie, K. J. (2017). Enhancing treatment effectiveness through social modeling: A pilot study. Psychol Health, 32, 626-637. doi:10.1080/08870446.2017.1293056

Faasse, K., Yeom, B., Parkes, B., Kearney, J., & Petrie, K. J. (2018). The Influence of Social Modeling, Gender, and Empathy on Treatment Side Effects. Annals of Behavioral Medicine, 52, 560-570. doi:10.1093/abm/kax025

Haug, S., Paz Castro, R., Wenger, A., & Schaub, M. P. (2021). A Mobile Phone–Based Life-Skills Training Program for Substance Use Prevention Among Adolescents: Cluster-Randomized Controlled Trial. JMIR Mhealth Uhealth, 9, e26951. doi: 10.2196/26951.

Hunter, T., Siess, F., & Colloca, L. (2014). Socially induced placebo analgesia: a comparison of a pre-recorded versus live face-to-face observation. Eur J Pain, 18, 914-22. doi: 10.1002/j.1532-2149.2013.00436.x

Jones, K. M., Bhattacharjee, R., Krishnamurthi, R., Blanton, S., Barker-Collo, S., Theadom, A., et al. (2018). Determining the feasibility and preliminary efficacy of a stroke instructional and educational DVD in a multinational context: a randomized controlled pilot study. Clin Rehabil., 32, 1086-1097. doi:10.1177/0269215518777565.

Karekla, M., Nikolaou, P., & Merwin, R. M. (2022). Randomized Clinical Trial Evaluating AcceptME—A Digital Gamified Acceptance and Commitment Early Intervention Program for Individuals at High Risk for Eating Disorders. Journal of Clinical Medicine, 11, 1775. doi:10.3390/jcm11071775.

Ledoux, T., Robinson, J., Baranowski, T., & O'Connor, D. P. (2018). Teaching Parents About Responsive Feeding Through a Vicarious Learning Video: A Pilot Randomized Controlled Trial. Health Education & Behavior, 45, 229-237. doi:10.1177/1090198117712332.

Levy, R. L., Langer, S. L., van Tilburg, M. A. L., Romano, J. M., Murphy, T. B., Walker, L. S., et al. 2017). Brief telephone-delivered cognitive behavioral therapy targeted to parents of children with functional abdominal pain: a randomized controlled trial. Pain, 158, 618-628. doi:10.1097/j.pain.0000000000000800

Maddison, R., Prapavessis, H., Armstrong, G. P., & Hill, C. (2008). A modeling intervention in heart failure. Ann Behav Med, 36, 64-69. doi:10.1007/s12160-008-9050-y.

Massouh, S. R., Steele, T. M., Alseth, E. R., & Diekmann, J. M. (1989). The effect of social learning intervention on metabolic control of insulin-dependent diabetes mellitus in adolescents. Diabetes Educ, 15, 518-521.doi:10.1177/014572178901500609

Meeuwis, S., Wasylewski, M., Bajcar, A., Bieniek, H., Adamczyk, W., Honcharova, S., Di Nardo, M., Mazzoni, G., Babel, P. (2023). Learning pain from others: a systematic review and meta-analysis of studies on placebo hypoalgesia and nocebo hyperalgesia induced by observational learning. *Pain*, Publish Ahead of Print.

Perry, R. P., Lebow, M. D., & Buser, M. M. (1979). An exploration of obese observational learning in modifying selected eating responses of obese children. International Journal of Obesity, 3, 193-199.

Raghuraman, N., Wang, Y., Schenk, L. A., Furman, A. J., Tricou, C., Seminowicz, D. A. & Colloca, L. (2019). Neural and behavioral changes driven by observationally-induced hypoalgesia. Sci Rep, 9, 19760. doi:10.1038/s41598-019-56188-2

Schenk, L. A. & Colloca, L. (2020). The neural processes of acquiring placebo effects through observation. NeuroImage, 209, 116510. https://doi.org/10.1016/j.neuroimage.2019.116510

Schwartz, M., Fischer, L. M., Bläute, C., Stork, J., Colloca, L., Zöllner, C. & Klinger, R. (2022a). Observing treatment outcomes in other patients can elicit augmented placebo effects on pain treatment: a double-blinded randomized clinical trial with patients with chronic low back pain. Pain, 163, 1313-1323. doi:10.1097/j.pain.0000000000002513

Swider, K., & Babel, P. (2013). The effect of the sex of a model on nocebo hyperalgesia induced by social observational learning. Pain, 154, 1312-1317. doi:10.1016/j.pain.2013.04.001

Świder, K., & Bąbel, P. (2016). The Effect of the Type and Colour of Placebo Stimuli on Placebo Effects Induced by Observational Learning. PLOS ONE, 11, e0158363. doi:10.1371/journal.pone.0158363

Tu, Y., Park, J., Ahlfors, S. P., Khan, S., Egorova, N., Lang, C., Cao, J., & Kong, J. (2019). A neural mechanism of direct and observational conditioning for placebo and nocebo responses. Neuroimage, 184, 954-963. doi:10.1016/j.neuroimage.2018.10.020

Vögtle, E., Barke, A., & Kröner-Herwig, B. (2013). Nocebo hyperalgesia induced by social observational learning. Pain, 154, 1427-1433. doi:10.1016/j.pain.2013.04.041

Vögtle, E., Kröner-Herwig, B., & Barke, A. (2016). Nocebo hyperalgesia: contributions of social observation and body-related cognitive styles. Journal of Pain Research, 9, 241-249. doi:10.2147/jpr.S96228

Vögtle, E., Kröner-Herwig, B., & Barke, A. (2019). Nocebo Hyperalgesia can be Induced by the Observation of a Model Showing Natural Pain Expressions. Clinical Journal of Pain, 35, 737-743. doi:10.1097/ajp.0000000000000734

Whittaker, R., Dorey, E., Bramley, D., Bullen, C., Denny, S., Elley, C. R. et al.(2011). A theory-based video messaging mobile phone intervention for smoking cessation: randomized controlled trial. Journal of Medical Internet Research, 13, e10. doi:10.2196/jmir.1553

Zhang, H., Zhou, L., Wei, H., Lu, X., & Hu, L. (2017). The sustained influence of prior experience induced by social observation on placebo and nocebo responses. Journal of Pain Research, 10, 2769-2780. doi:10.2147/jpr.S147970
